# Supplementary material for: Myopia prevalence and ocular biometry in children and adolescents at different altitudes: a cross-sectional study in Chongqing and Tibet, China
Source: BMJ Open. 2024 May 1;14(5):e078018. doi: 10.1136/bmjopen-2023-078018 (PMC11086200; doi:10.1136/bmjopen-2023-078018)
Supplement: Supplementary data [file bmjopen-2023-078018supp001.pdf]

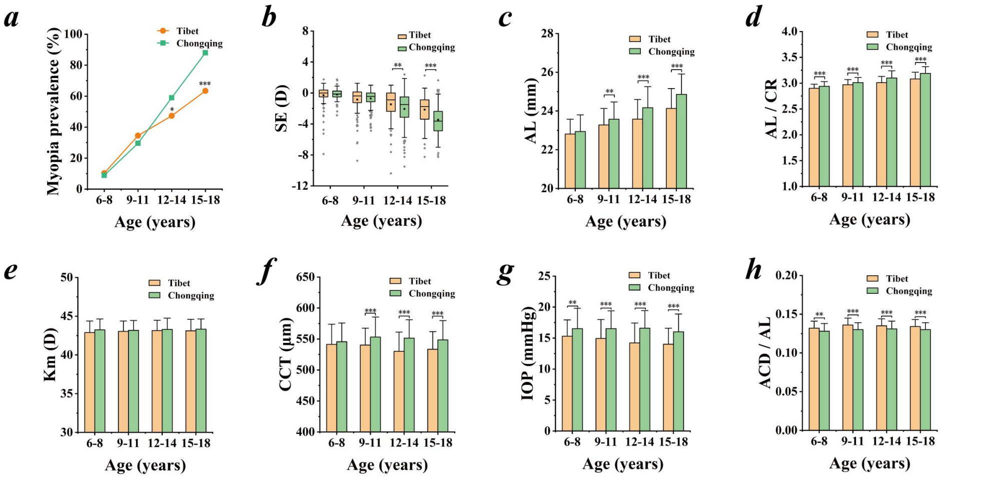

Figure S1 Comparison of myopia prevalence (a), SE (b), AL (c), AL/CR (d), Km (e), CCT (f), IOP (g), and ACD/AL (h) in Tibetan and Chongqing children and adolescents by age groups. SE, spherical equivalent; D, diopter; AL, axial length; AL/CR, AL to corneal radius of curvature ratio; Km, mean curvature power of the cornea; CCT, corneal thickness at the apex;IOP, intraoccular pressure; ACD, internal anterior chamber depth.

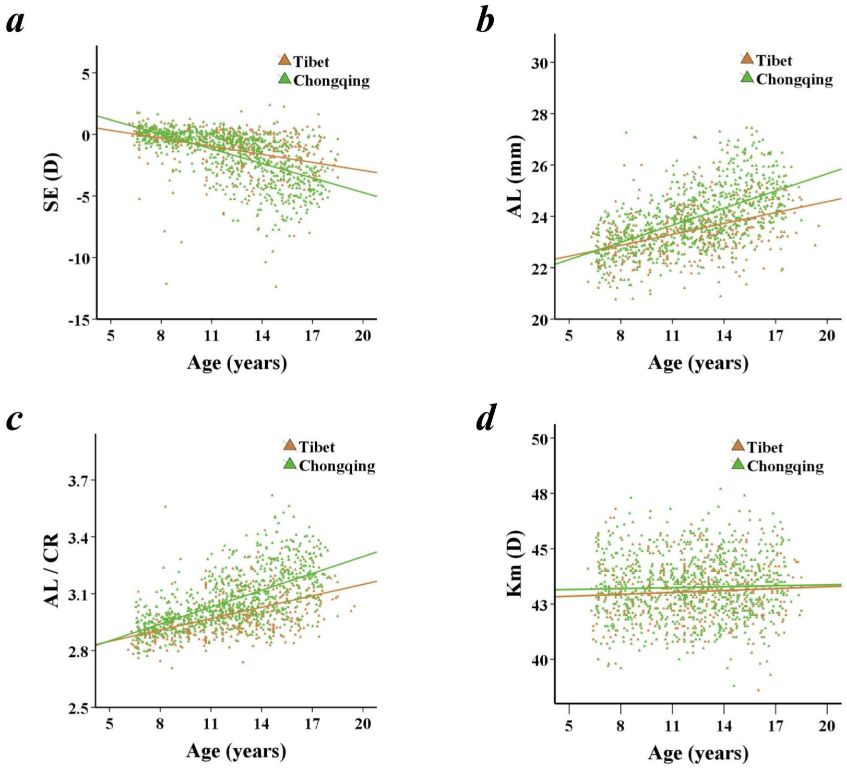

Figure S2 Linear regression model of SE (a), AL (b), AL/CR (c) and Km (d) with age. SE, spherical equivalent; D, diopter; AL, axial length; AL/CR, AL to corneal radius of curvature ratio; Km, mean curvature power of the cornea.

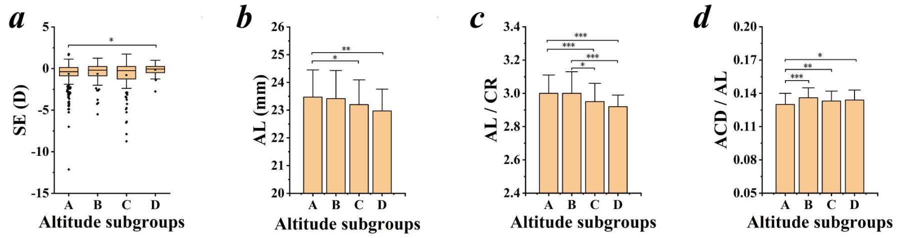

Figure S3 Comparison of SE (a), AL (b), AL/CR (c) and ACD/AL (d) in altitude subgroups of primary school students. SE, spherical equivalent; D, diopter; AL, axial length; AL/CR, AL to corneal radius of curvature ratio; ACD, internal anterior chamber depth. Group A, with an average altitude of 325 meters; Group B, with an average altitude of 2,300 meters; Group C, with an average altitude of 3,250 and 3,170 meters, Group D, with an average altitude of 3,870 meters.
