# Supplementary material for: Myopia prevalence and ocular biometry in children and adolescents at different altitudes: a cross-sectional study in Chongqing and Tibet, China
Source: BMJ Open. 2024 May 1;14(5):e078018. doi: 10.1136/bmjopen-2023-078018 (PMC11086200; doi:10.1136/bmjopen-2023-078018)
Supplement: Author's manuscript [file bmjopen-2023-078018.draft_revisions.pdf]

BMJ Open is committed to open peer review. As part of this commitment we make the peer review history of every article we publish publicly available.

When an article is published we post the peer reviewers' comments and the authors' responses online. We also post the versions of the paper that were used during peer review. These are the versions that the peer review comments apply to.

The versions of the paper that follow are the versions that were submitted during the peer review process. They are not the versions of record or the final published versions. They should not be cited or distributed as the published version of this manuscript.

BMJ Open is an open access journal and the full, final, typeset and author-corrected version of record of the manuscript is available on our site with no access controls, subscription charges or pay-per-view fees (http://bmjopen.bmj.com).

If you have any questions on BMJ Open's open peer review process please email info.bmjopen@bmj.com

BMJ Open

Myopia Prevalence and Ocular Biometry in Children and Adolescents at Different Altitudes: A Cross-sectional Study in Chongqing and Tibet, China

Journal:	*BMJ Open*
Manuscript ID	bmjopen-2023-078018
Article Type:	Original research
Date Submitted by the Author:	10-Aug-2023
Complete List of Authors:	Xiang, Yongguo; The First Affiliated Hospital of Chongqing Medical University; Chongqing Medical University<br>Cheng, Hong; The First Affiliated Hospital of Chongqing Medical University; Chongqing Medical University<br>Sun, Kexin; The First Affiliated Hospital of Chongqing Medical University; Chongqing Medical University<br>Zheng, Shijie; The First Affiliated Hospital of Chongqing Medical University; Chongqing Medical University<br>Du, Miaomiao; The First Affiliated Hospital of Chongqing Medical University; Chongqing Medical University<br>Gao, Ning; The First Affiliated Hospital of Chongqing Medical University; Chongqing Medical University<br>Zhang, Tong; Chongqing Medical University<br>Yang, Xin; The First Affiliated Hospital of Chongqing Medical University<br>Xia, Jiuyi; The First Affiliated Hospital of Chongqing Medical University<br>Huang, Rongxi; Chongqing People's Hospital<br>Wan, Wenjuan; The First Affiliated Hospital of Chongqing Medical University; Chongqing Medical University<br>Hu, Ke; The First Affiliated Hospital of Chongqing Medical University; Chongqing Medical University
Keywords:	EPIDEMIOLOGIC STUDIES, Paediatric ophthalmology < OPHTHALMOLOGY, Medical ophthalmology < OPHTHALMOLOGY, Corneal and external diseases < OPHTHALMOLOGY

SCHOLARONE™
Manuscripts

I, the Submitting Author has the right to grant and does grant on behalf of all authors of the Work (as defined in the below author licence), an exclusive licence and/or a non-exclusive licence for contributions from authors who are: i) UK Crown employees; ii) where BMJ has agreed a CC-BY licence shall apply, and/or iii) in accordance with the terms applicable for US Federal Government officers or employees acting as part of their official duties; on a worldwide, perpetual, irrevocable, royalty-free basis to BMJ Publishing Group Ltd ("BMJ") its licensees and where the relevant Journal is co-owned by BMJ to the co-owners of the Journal, to publish the Work in this journal and any other BMJ products and to exploit all rights, as set out in our licence.

The Submitting Author accepts and understands that any supply made under these terms is made by BMJ to the Submitting Author unless you are acting as an employee on behalf of your employer or a postgraduate student of an affiliated institution which is paying any applicable article publishing charge ("APC") for Open Access articles. Where the Submitting Author wishes to make the Work available on an Open Access basis (and intends to pay the relevant APC), the terms of reuse of such Open Access shall be governed by a Creative Commons licence – details of these licences and which Creative Commons licence will apply to this Work are set out in our licence referred to above.

Other than as permitted in any relevant BMJ Author's Self Archiving Policies, I confirm this Work has not been accepted for publication elsewhere, is not being considered for publication elsewhere and does not duplicate material already published. I confirm all authors consent to publication of this Work and authorise the granting of this licence.

For peer review only

Myopia Prevalence and Ocular Biometry in Children and Adolescents at Different Altitudes: A Cross-sectional Study in Chongqing and Tibet, China

Yongguo Xiang[1,2]*, Hong Cheng[1,2]*, Kexin Sun[1,2], Shijie Zheng[1,2], Miaomiao Du[1,2], Ning Gao[1,2], Tong Zhang[2], Xin Yang[1], Jiuyi Xia[1], Rongxi Huang[3], Wenjuan Wan[1,2]* and Ke Hu[1,2]*

1 The First Affiliated Hospital of Chongqing Medical University, Chongqing Key Laboratory of Ophthalmology, Chongqing Eye Institute, Chongqing Branch (Municipality Division) of National Clinical Research Center for Ocular Diseases, Chongqing, People's Republic of China

2 Chongqing Medical University, Chongqing, People's Republic of China

3 Chongqing General Hospitoal, Chongqing, People's Republic of China

*These authors contributed equally to this work

Corresponding Author: Wenjuan Wan, wanwenjuancqums@163.com, and Ke Hu, 42222@qq.com

Objective: To investigate the differences in myopia prevalence and ocular biometry in children and adolescents in Chongqing and Tibet, China.

Methods: This school-based cross-sectional study was conducted among children and adolescents aged 6-18 years in Chongqing, a low-altitude region, and in Qamdo, a high-altitude region of Tibet. All participants underwent uncorrected visual acuity (UCVA) assessment, non-cycloplegic refraction, axial length (AL) measurement, intraocular pressure (IOP) measurement, and corneal tomography. And the participants were grouped according to age (6-8, 9-11, 12-14, and 15-18 years group), and altitude of location [primary school students: Group A (average altitude: 325 meters), Group B (average altitude: 2,300 meters), Group C (average altitude: 3,250 and 3,170 meters), Group D (average altitude: 3,870 meters)].

For peer review only - http://bmjopen.bmj.com/site/about/guidelines.xhtml

Results: A total of 448 participants in Qamdo, Tibet, and 748 participants in Chongqing were enrolled in this study. There was no statistical difference in mean age (12.09 ± 3.15 vs. 12.2 ± 3.10, p = 0.549) and sex distribution (males, 50.4% vs. 47.6%, p = 0.339) between the two groups. The Tibet group presented greater spherical equivalent (SE ,-1.21 ± 1.82 vs. -1.65 ± 2.05, p < 0.001), shorter AL (23.45 ± 1.02 vs. 23.92 ± 1.19, p < 0.001), lower prevalence of myopia (39.7% vs. 47.6%, p = 0.008), and flatter mean curvature power of the cornea (Km, 43.06 ± 1.4 vs. 43.26 ± 1.36, p = 0.014) than the Chongqing group. Further analysis based on age subgroups revealed that the Tibet group had a lower prevalence of myopia and higher SE in the 12-14, and 15-18 years old groups, shorter AL in the 9-11, 12-14, and 15-18 years old groups, and lower AL to corneal radius of curvature ratio (AL/CR) in all age subgroups compared to the Chongqing group, while Km was similar between the two groups in each age subgroup. Simple linear regression analysis showed that SE decreased with age in both the Tibet and Chongqing groups, with the Tibet group exhibiting a slower rate of decrease (p < 0.001). AL and AL/CR increased with age in both the Tibet and Chongqing groups, but the rate of increase was slower in the Tibet group (p < 0.001 of both). Multiple linear regression analysis revealed that AL had the greatest effect on SE in both groups, followed by Km. In addition, the children and adolescents in Tibet presented thinner corneal thickness (CCT, p < 0.001), smaller white to white distance (WTW, p < 0.001), lower IOP (p < 0.001), and deeper anterior chamber depth (ACD, p = 0.015) than in Chongqing. Comparison of altitude subgroups showed that the prevalence of myopia (p = 0.002), AL (p = 0.001), and

For peer review only - http://bmjopen.bmj.com/site/about/guidelines.xhtml

AL/CR ($p < 0.001$) of children at different altitudes were statistically different but the Km ($p = 0.189$) were similar. The highest altitude, Tengchen County, exhibited the lowest prevalence of myopia among children, and the mean AL also decreased with increasing altitude.

Conclusions: Myopia prevalence in Tibet was comparable to that in Chongqing for students aged 6-8 and 9-11 years but was lower and myopia progressed more slowly for students aged 12-14 and 15-18 years than in Chongqing, and AL was the main contributor for this difference, which may be related to higher ultraviolet radiation exposure and lower IOP in children and adolescents at high altitude in Tibet. Differences in AL and AL/CR between Tibet and Chongqing children and adolescents manifested earlier than in SE, underscoring the importance of AL measurement in myopia screening.

Key words: prevalence of myopia, ocular biometry, children and adolescents, altitude, ultraviolet radiation

BACKGROUND

It is reported that approximately 400 million people worldwide live at altitudes above 1,500 meters[1]. The Qinghai-Tibet Plateau is the highest geographical unit in the world. Qamdo is located in the eastern part of the Qinghai-Tibet Plateau, with an average altitude of more than 3,500 meters. It is characterized by low atmospheric pressure, hypoxia, cold and dry weather, and strong ultraviolet radiation (UVR)[2].

Previous study have shown that UVR intensity increases by 10-12% for every 1000

For peer review only - http://bmjopen.bmj.com/site/about/guidelines.xhtml

meters of altitude. And approximately 90-99% of the solar UVR energy that reaches the earth's surface is UVA (315-400 nm wavelength), whereas only 1-10% is UVB (280-315nm wavelength)[3]. The cornea sits at the anterior aspect of the eye and, like the skin, is highly exposed to UVR. Torii et al.[4] found that violet light (VL, 360-400 nm wavelength) suppresses myopia progression by upregulating the myopia suppressive gene EGR1. Kobashi et al.[5] reported that there is a small amount of riboflavin in the human corneal stroma, and the ex vivo corneal stiffness significantly increases after VL (375 nm wavelength) irradiation based on physiological riboflavin. Epidemiological data show that the prevalence of keratoconus, pterygium, dry eye disease, cataract, and strabismus is significantly higher in the highlands than in the lowlands[2][6][7][8][9], and the cornea of the highlanders is thinner than that of the lowlanders[10][11]. Our previous study also found that the prevalence of myopia among children and adolescents in Tibet was lower than that in the plains[12]. However, it is unclear whether ocular biometry is associated with myopia prevalence between high and low altitude populations with different levels of UVR exposure.

The axial length (AL) and AL to corneal radius of curvature ratio (AL/CR) have been reported to be strongly correlated with ocular refraction, and play an important role in the screening and monitoring of myopia[13]. Cycloplegic refraction is considered the gold standard for measuring refractive errors, but limited by the fact that mydriatic drops can induce mydriasis and cycloplegia[14] Consequently, noncycloplegic refraction remains a dependable evaluation index in large scale myopia investigations.

For peer review only - http://bmjopen.bmj.com/site/about/guidelines.xhtml

In this study, we compared the prevalence of myopia and ocular biometry in children and adolescents in Chongqing and Qamdo, Tibet, and attempted to explore the effect of altitude on ocular biometry.

MATERIALS AND METHODS

This school-based cross-sectional study was conducted among children and adolescents aged 6-18 years in Chongqing, a low-altitude region, and in Qamdo, a high-altitude region of Tibet. The Ethics Committee of the First Affiliated Hospital of Chongqing Medical University approved this study and it was conducted in accordance with the tenets of the Declaration of Helsinki. At least one parent or legal guardian of each participant was informed about the study and signed an informed consent form.

Study Population

This study selected Karo district, Tengchen county, Markam county, and Drayab county in Qamdo, Tibet, as well as Shapingba district and Banan district in Chongqing, as the investigation sites. A stratified sampling method was employed to select students ranging from the first grade through the senior second level at each investigation site. Ultimately, a total of 480 students from Qamdo, Tibet, and 944 students from Chongqing were included in this school-based study. The specifics of the sample inclusion are presented in Table 1.

Methods

All participants underwent a standardized ophthalmic examination. Uncorrected

For peer review only

For peer review only - http://bmjopen.bmj.com/site/about/guidelines.xhtml

visual acuity (UCVA) was assessed and recorded in LogMAR scores by using the standard logarithmic visual acuity chart at 5 meters. Noncycloplegic refraction was performed with an autorefractor (Supore, China). Axial length (AL) was measured with the AL-scan (NIDEK, Japan). Intraocular pressure (IOP) was assessed with a non-contact tonometer (NIDEK, Japan). Corneal and anterior chamber parameters were measured with the Pentacam AXL (Oculus Optikgeräte GmbH, Wetzlar, Germany). The quality specification section on the output graph was used to assess the quality, with an "OK" reading indicating an acceptable quality. All examinations were performed by skilled ophthalmologists. At least three measurements were made for each eye and averaged, but only one eye of each participant was randomly selected for statistical analysis. Spherical equivalent (SE) equals diopter (D) of spherical power plus 1/2 diopter (D) of cylindrical power. Myopia was defined as SE <-0.5D and UCVA >0 logMAR.

The investigated indices were as follows: UCVA, SE, AL, IOP, AL to corneal radius of curvature ratio (AL/CR); mean curvature power of the cornea within the central 3-mm circle (Km), astigmatism of the front surface (Astig. F); astigmatism of the back surface (Astig. B); corneal thickness at the apex (CCT); white to white distance (WTW); internal anterior chamber depth (ACD).

The participants were grouped according to age (6-8 years group, 9-11 years group, 12-14 years group, and 15-18 years group), and altitude of location [primary school students: Group A (Shapingba and Banan district, average altitude: 325 meters), Group B (Qucainka township, average altitude: 2,300 meters), Group C (Karo district

For peer review only - http://bmjopen.bmj.com/site/about/guidelines.xhtml

and Drayab county, average altitude: 3,250 and 3,170 meters, respectively), Group D (Xiexiong Township, average altitude: 3,870 meters)].

Statistical Analysis

The statistical analysis was performed with SPSS version 26 (IBM Corp. in Armonk, NY, USA). The chi-squared test was utilized to compare categorical variables, while the independent samples t-test and analysis of variance (ANOVA) were employed to compare continuous variables. Dunn-Bonferroni post-hoc test was conducted for pairwise comparisons. ANCOVA test was applied to compare IOP, WTW, and ACD between the Tibet and Chongqing groups, with adjustments made for CCT or AL. Simple linear regression analysis was performed to examine the relationship between SE, AL, AL/CR, and age. Multiple linear regression analysis was conducted to explore the relationship between AL, Km, and SE. The significance level was set at $p < 0.05$.

RESULTS

Thirty-two students from Tibet and 181 students from Chongqing were excluded due to lack of qualified ocular biometric results, and 15 students from Chongqing were excluded for wearing corneal contact lenses. Finally, a total of 448 students in Tibet and 748 students in Chongqing were enrolled in this study. The distribution of participants by gender and age is presented in Table 2. And there were no statistical difference observed in the mean age (12.09 ± 3.15 vs. 12.2 ± 3.10, $p = 0.549$, Table 3) and sex distribution (males, 50.4% vs. 47.6%, $p = 0.339$, Table 3) between the two groups.

For peer review only - http://bmjopen.bmj.com/site/about/guidelines.xhtml

Comparison between the Tibet group and the Chongqing group

As shown in Table 3, the Tibet group presented a lower prevalence of myopia (39.7% vs. 47.6%, p = 0.008), greater SE (-1.21 ± 1.82 vs. -1.65 ± 2.05, p < 0.001), shorter AL (23.45 ± 1.02 vs. 23.92 ± 1.19, p < 0.001), flatter Km (43.06 ± 1.4 vs. 43.26 ± 1.36, p = 0.014), and smaller AL/CR (2.99 ± 0.13 vs.3.06 ± 0.15, p < 0.001) than the Chongqing group, while the astigmatism of the anterior (1.17 ± 0.68 vs. 1.24 ± 0.68, p = 0.086) and posterior (0.34 ± 0.14 vs. 0.36 ± 0.14, p = 0.175) corneal surface was similar between the two groups. Further analysis based on age subgroups revealed that the Tibet group had a lower prevalence of myopia and higher SE in the 12-14, and 15-18 years old groups, shorter AL in the 9-11, 12-14, and 15-18 years old groups, and lower AL/CR in all age subgroups compared to the Chongqing group, while Km was similar between the two groups in each age subgroup (Table 3, Supplemental Figure 1a, 1b, 1c and 1d).

The children and adolescents in Tibet exhibited thinner CCT (535.78 ± 30.16 vs. 550.01 ± 30.8, p < 0.001), smaller WTW (11.62 ± 0.38 vs. 11.71 ± 0.37, p < 0.001, after adjustment for AL, p = 0. 039), deeper ACD (3.15 ± 0.26 vs. 3.11 ± 0.29, p = 0.015, after adjustment for AL, p < 0.001), and lower IOP (14.59 ± 2.96 vs. 16.43 ± 2.95, p < 0.001, after adjustment for CCT, p < 0.001) than those in Chongqing. Subgroup analysis based on age revealed similar differences in CCT, except for the 6-8 years age group (Supplemental Fig. 1e). Furthermore, after adjusting for AL, the ACD was found to be deeper in the Tibet group across all age subgroups, while the WTW remained similar.

For peer review only - http://bmjopen.bmj.com/site/about/guidelines.xhtml

Regression analysis

The simple linear regression analysis showed that SE decreased with age in both the Tibet (R^2 = 0.140, $p < 0.001$) and Chongqing (R^2 = 0.352, $p < 0.001$) groups, with the Tibet group exhibiting a slower rate of decrease (B: -0.217 vs. -0.393, $p < 0.001$, Supplemental Figure 2a). Additionally, AL and AL/CR increased with age in both Tibet (AL: R2 = 0.192, $p < 0.001$; AL/CR: R^2 = 0.253, $p < 0.001$) and Chongqing (AL: R^2 = 0.335, $p < 0.001$; AL/CR: R^2 = 0.391, $p < 0.001$) groups, but the rate of increase was slower in the Tibet group (B: AL: 0.143 vs. 0.223, $p < 0.001$, Figure 2a; AL/CR: 0.02 vs. 0.03, $p < 0.001$, Figure 2c). Conversely, there was no linear relationship between Km and age for both the Tibet (R^2 = 0.004, p = 0.173) and Chongqing (R^2 = 0.001, p = 0.377, Figure 2d) groups. Furthermore, the multiple linear regression analysis revealed that AL had the greatest effect on SE in both groups, followed by Km (Table 4).

Comparison of children among altitude subgroups

As shown in Table 5, there was no statistical difference in the mean age (p = 0.557) and sex distribution (p = 0.979) among the altitude subgroups. Comparison of altitude subgroups showed that the prevalence of myopia (p = 0.002, Supplemental Figure 3a), AL (p = 0.001, Figure 3b), and AL/CR ($p < 0.001$, Figure 3c) of children at different altitudes were statistically different but the Km (p = 0.189) were similar. Notably, the highest altitude, Tengchen County, exhibited the lowest prevalence of myopia among children, and the mean AL value also decreased with increasing altitude.

DISCUSSION

For peer review only - http://bmjopen.bmj.com/site/about/guidelines.xhtml

In this school-based cross-sectional study, we conducted a comparison of the prevalence of myopia and ocular biometry among students aged 6-18 years in Chongqing and Qamdo, Tibet. Our findings indicate that the prevalence of myopia and SE were comparable between the two locations during the age groups of 6-8 years and 9-11 years. However, we observed a lower prevalence of myopia and a higher SE in Tibet compared to Chongqing among students aged 12-14 years and 15-18 years. This discrepancy may be attributed to the slower progression of myopia with advancing age in the Tibetan population. The Tibetan students exhibited a shorter AL within the 9-11, 12-14, and 15-18 years old age groups, as well as lower AL/CR across all age subgroups in comparison to the students from Chongqing. However, the Km values were found to be similar between the two locations within each respective age subgroup.

Myopia is one of the most common eye disorders globally, caused by both environmental and genetic risk factors[15]. And the increasing prevalence of myopia can be largely explained by increased educational pressures and reductions in the amount of time that children spend outdoors[16]. Qian et al.[17] and Wang et al.[12] have reported that Tibetan children and adolescents at high altitudes had a lower prevalence of myopia than their counterparts at low altitudes. In line with their findings, our study also revealed that children and adolescents in Tibet presented a lower overall prevalence of myopia, shorter AL, higher SE, and slower myopia progression in comparison to individuals in Chongqing. Furthermore, the prevalence of myopia among children and adolescents in Tibet was found to be lower than

For peer review only - http://bmjopen.bmj.com/site/about/guidelines.xhtml

previous reports in other plain regions of China[18][19][20]. Interestingly, the prevalence of myopia and SE of children aged 6-8 and 9-11 years in Tibet in our study were similar to those in Chongqing, and similar findings were reported by Wu et al[21], suggesting that myopia is widespread among children in high altitude regions and that myopia prevention and control in high altitude areas of China are equally challenging.

In recent years, much research evidence suggests that outdoor activity in bright light conditions is a protective factor against myopia[15][16]. The mechanism for this protective effect appears to be related to an increased release of dopamine stimulated by visible light (400-700 nm wavelength), which inhibits increased axial elongation[22]. In addition, Tsubota's team reported that VL, with wavelengths from 360 to 400 nm, can effectively suppress myopia progression in chicks, mice, and humans[4][23][24]. Qamdo is located in the eastern part of the Qinghai-Tibet Plateau, with an average altitude of more than 3,500 meters, and the UVR intensity, especially UVA intensity (315-400 nm wavelength), is stronger in Qamdo than that in Chongqing due to the increase in UVR intensity with altitude[3]. Additionally, Qamdo experiences an average daily sunshine duration of 7.18 hours, approximately twice the duration observed in Chongqing, which amounts to 3.54 hours. (https://www.weather2visit.com/). The results of our study indicated that children and adolescents in Tibet exhibited shorter AL and slower AL elongation in comparison to their counterparts in Chongqing. Furthermore, our analysis of altitude subgroups revealed a decrease in the mean value of AL with increasing altitude. Although

For peer review only - http://bmjopen.bmj.com/site/about/guidelines.xhtml

information regarding outdoor activities was not collected in this study for both groups, our previous research demonstrated that children and adolescents in Tibet spend more time outdoors than those in Chongqing[12], which may be synergistic with stronger UVR intensity and longer average daily sunshine in Tibet in slowing myopia progression.

The findings from the multiple linear regression analysis indicated that the AL and Km were the primary determinants of SE. Notably, the Km remained consistent with age in both Tibet and Chongqing children and adolescents. Consequently, the AL emerged as the primary factor contributing to the disparity in myopia progression between the two locations. Moreover, the differences in AL between Tibet and Chongqing children and adolescents manifested earlier than those in SE, underscoring the importance of AL measurement in myopia screening. Corneal curvature as well as ACD also contribute to myopic progression[25]. In the case of children and adolescents in Tibet, the mean corneal curvature was found to be flatter compared to those in Chongqing, albeit with a minimal impact on the difference in myopia. However, after adjusting for AL, it was observed that the ACD in Tibetan children and adolescents was deeper than that in Chongqing. This suggested that the lens in Tibetan individuals may be either flatter or positioned more posteriorly, resulting in a stronger compensatory effect on the elongation of AL[26][27]. However, regrettably, the absence of lens curvature data is attributed to the constraints of the examination equipment, and the investigation into the association between lens curvature and myopia will be undertaken in subsequent research.

For peer review only - http://bmjopen.bmj.com/site/about/guidelines.xhtml

The mechanism of myopia development was considered to be associated with scleral matrix remodeling[28]. And scleral matrix remodeling has been shown to contribute to the biomechanical susceptibility of the sclera to accommodation-induced IOP fluctuations, resulting in reduced scleral thickness, AL elongation, and axial myopia. The rise in IOP can increase the burden of scleral stretching and cause axial lengthening[29]. In addition, lowering IOP was also considered a potential approach for controlling the progression of high myopia[30]. Several clinical studies have examined the association between IOP and the progression of myopia, but the results were inconsistent[29]. In our study, we observed that IOP levels were consistently higher in children and adolescents residing in Chongqing compared to those in Tibet, across all age subgroups. In a cross-sectional, multicenter, population-based study including 284,937 participants conducted by Liu et al.**Error! Reference source not found.**, they also reported that IOP was significantly higher in low-altitude populations than in high-altitude populations. This disparity persisted after adjusting for CCT, which may suggest a potential link between elevated IOP and myopia. However, further longitudinal studies are required to establish a causal relationship between IOP and AL elongation. The underlying reasons for the observed disparity in IOP between these two populations remain unclear.

In our study, it was observed that Tibetan children and adolescents exhibited a thinner corneal thickness compared to their counterparts in Chongqing. This finding aligns with previous research conducted by Amit et al. [10] and Patyal et al. [11], who also reported a thinner CCT in individuals residing at higher altitudes. Additionally,

For peer review only - http://bmjopen.bmj.com/site/about/guidelines.xhtml

our study revealed that the CCT of Tibetan children and adolescents was thinner than what was previously observed in studies conducted at lower altitudes[31][32][33]. This discrepancy may be attributed to variations in UVR exposure levels, as evidenced by the study conducted by Asharlous et al.[34], which found that welders, who are exposed to long-term UVR, exhibited a significantly thinner cornea compared to healthy controls. Hence, it is plausible to consider long-term UVR exposure as a potential contributor to corneal thinning.

Our current study had several limitations. Firstly, the limited sample size employed in this study may hinder a comprehensive evaluation of the ocular biometric characteristics in children and adolescents residing in the plateau region. Secondly, the accommodative system of children is vigorous, which may introduce inaccuracies in noncycloplegic refraction, potentially resulting in an overestimation of myopia. Lastly, this study primarily examined variations in ocular biometry among children and adolescents living at different altitudes. However, further research is needed to explore the potential correlation between ocular biometry and behavioral habits. In addition, cross-sectional studies cannot accurately assess the progression of myopia. In the future, we will conduct prospective studies to provide more reliable conclusions.

In conclusion, this study investigated the prevalence of myopia and ocular biometry of children and adolescents aged 6-18 years in Chongqing and Qamdo, Tibet. Students aged 12-18 years had a lower prevalence of myopia and greater SE in Tibet than in Chongqing, but no discernible difference was observed among students

For peer review only - http://bmjopen.bmj.com/site/about/guidelines.xhtml

aged 6-11 years, indicating a comparatively slower progression of myopia in Tibetan students, which may be related to higher UVR exposure, flatter corneas, and lower IOP in Tibetan children and adolescents. In addition, we found that the Tibetan population had thinner corneas than the Chongqing population, which may be associated with long-term UVR exposure.

For peer review only

Funding

This work was supported by the National Natural Science Foundation of China (Grant nos. 81870650, 82000883, 81900885 and 81970832), the Project Foundation of Chongqing Science and Technology Commission of China (Grant nos. CSTC2021jscx-gksb-N0017, cstc2020jcyj-msxmX0829, CSTB2022NSCQ-MSX1561, and cstc2021jcyj-msxmX0967) and Chongqing Talent Plan "Contract program" (Grant no. cstc2022ycjh-bgzxm0121).

Competing interests

The authors declare no competing interests.

Author contributions

Study conception and design: YGX, HC, WJW and KH; data collection and statistics: YGX, HC, KXS, SJZ, MMD, NG, TZ, XY, JYX and RXH; data interpretation: YGX and HC; writing-original draft: YGX; writing—review and editing: WW and KH. All authors contributed to the article and approved the submitted version.

Ethics approval and consent to participate

The study adhered to the Declaration of Helsinki or relevant guidelines and

For peer review only - http://bmjopen.bmj.com/site/about/guidelines.xhtml

regulations, and was approved by the ethical review committee of the First Affiliated Hospital of Chongqing Medical University, Chongqing, China. Written informed consent to participate in this study was provided by the participants/next of kin.

Consent for publication

Not applicable.

Availability of data and materials

The datasets generated and/or analyzed during the current study are available from the corresponding author upon reasonable request.

Acknowledgments

We are indebted to KH and WJW from the First Affiliated Hospital of Chongqing Medical University, Chongqing, China for their help in supervising this manuscript.

For peer review only - http://bmjopen.bmj.com/site/about/guidelines.xhtml

References

[1] Cohen JE, Small C. Hypsographic demography: the distribution of human population by altitude. Proc Natl Acad Sci U S A. 1998;95(24):14009-14014. doi:10.1073/pnas.95.24.14009

[2] Wang GQ, Bai ZX, Shi J, Luo S, Chang HF, Sai XY. Prevalence and risk factors for eye diseases, blindness, and low vision in Lhasa, Tibet. Int J Ophthalmol. 2013;6(2):237-241. Published 2013 Apr 18. doi:10.3980/j.issn.2222-3959.2013.02.24

[3] Narayanan DL, Saladi RN, Fox JL. Ultraviolet radiation and skin cancer. Int J Dermatol. 2010 Sep;49(9):978-86. doi: 10.1111/j.1365-4632.2010.04474.x. PMID: 20883261.

[4] Torii H, Kurihara T, Seko Y, Negishi K, Ohnuma K, Inaba T, Kawashima M, Jiang X, Kondo S, Miyauchi M, Miwa Y, Katada Y, Mori K, Kato K, Tsubota K, Goto H, Oda M, Hatori M, Tsubota K. Violet Light Exposure Can Be a Preventive Strategy Against Myopia Progression. EBioMedicine. 2017 Feb;15:210-219. doi: 10.1016/j.ebiom.2016.12.007. Epub 2016 Dec 16.

[5] Kobashi H, Yunoki S, Kato N, Shimazaki J, Ide T, Tsubota K. Evaluation of the Physiological Corneal Intrastromal Riboflavin Concentration and the Corneal Elastic Modulus After Violet Light Irradiation. Transl Vis Sci Technol. 2021;10(5):12. doi:10.1167/tvst.10.5.12

[6] Althomali TA, Al-Qurashi IM, Al-Thagafi SM, Mohammed A, Almalki M. Prevalence of keratoconus among patients seeking laser vision correction in Taif

For peer review only

For peer review only - http://bmjopen.bmj.com/site/about/guidelines.xhtml

area of Saudi Arabia. Saudi J Ophthalmol. 2018;32(2):114-118. doi:10.1016/j.sjopt.2017.11.003

[7] Guo B, Lu P, Chen X, Zhang W, Chen R. Prevalence of dry eye disease in Mongolians at high altitude in China: the Henan eye study. Ophthalmic Epidemiol. 2010;17(4):234-241. doi:10.3109/09286586.2010.498659

[8] Jiachu D, Jin L, Jiang F, et al. Prevalence and service assessment of cataract in Tibetan areas of Sichuan Province, China: population-based study. BMJ Open. 2019 Nov 3;9(11):e031337.

[9] He H, Fu J, Meng Z, Chen W, Li L, Zhao X. Prevalence and associated risk factors for childhood strabismus in Lhasa, Tibet, China: a cross-sectional, school-based study. BMC Ophthalmol. 2020;20(1):463. Published 2020 Nov 25. doi:10.1186/s12886-020-01732-2

[10] Amit A, Gaurav K, Vikas A, Ashok K, Harpreet AS, Shivani A. Evaluation of intraocular pressure and corneal thickness in individuals at high altitude area (10000 ft above sea level). Rom J Ophthalmol. 2019;63(3):217-221

[11] Patyal S, Arora A, Yadav A, Sharma VK. Corneal Thickness in Highlanders. High Alt Med Biol. 2017;18(1):56-60. doi:10.1089/ham.2016.0074

[12] Wang W, Xiang Y, Zhu L, et al. Myopia progression and associated factors of refractive status in children and adolescents in Tibet and Chongqing during the COVID-19 pandemic. Front Public Health. 2022;10:993728. Published 2022 Oct 13. doi:10.3389/fpubh.2022.993728

[13] Mu J, Zeng D, Fan J, et al. The accuracy of the axial length and axial

For peer review only - http://bmjopen.bmj.com/site/about/guidelines.xhtml

length/corneal radius ratio for myopia assessment among Chinese children. Front Pediatr. 2022;10:859944. Published 2022 Sep 6. doi:10.3389/fped.2022.859944

[14] Wilson S, Ctori I, Shah R, Suttle C, Conway ML. Systematic review and meta-analysis on the agreement of non-cycloplegic and cycloplegic refraction in children. Ophthalmic Physiol Opt. 2022 Nov;42(6):1276-1288. doi: 10.1111/opo.13022. Epub 2022 Aug 1. PMID: 35913773; PMCID: PMC9804580

[15] Baird PN, Saw SM, Lanca C, et al. Myopia. Nat Rev Dis Primers. 2020;6(1):99. Published 2020 Dec 17. doi:10.1038/s41572-020-00231-4

[16] Morgan IG, French AN, Ashby RS, et al. The epidemics of myopia: Aetiology and prevention. Prog Retin Eye Res. 2018;62:134-149. doi:10.1016/j.preteyeres.2017.09.004

[17] Qian X, Liu B, Wang J, et al. Prevalence of refractive errors in Tibetan adolescents. BMC Ophthalmol. 2018;18(1):118. Published 2018 May 11. doi:10.1186/s12886-018-0780-8

[18] Zhang J, Li Z, Ren J, et al. Prevalence of myopia: A large-scale population-based study among children and adolescents in weifang, china. Front Public Health. 2022;10:924566. Published 2022 Jul 25. doi:10.3389/fpubh.2022.924566

[19] Xu L, Zhuang Y, Zhang G, et al. Design, methodology, and baseline of whole city-million scale children and adolescents myopia survey (CAMS) in Wenzhou, China. Eye Vis (Lond). 2021;8(1):31. Published 2021 Aug 19. doi:10.1186/s40662-021-00255-1

[20] He X, Sankaridurg P, Naduvilath T, et al. Normative data and percentile curves

For peer review only - http://bmjopen.bmj.com/site/about/guidelines.xhtml

for axial length and axial length/corneal curvature in Chinese children and adolescents aged 4-18 years. Br J Ophthalmol. 2023;107(2):167-175. doi:10.1136/bjophthalmol-2021-319431

[21] Wu Q, Tian Q, Zhang X, Xu J, Tang G, Li R, Guo X, Xu Z, Feng J, Song J, Bi H. Prevalence of Refractive Error and Visual Acuity Among School Children in the Plateau Region of Qinghai, China. Int J Gen Med. 2021 Sep 16;14:5795-5805. doi: 10.2147/IJGM.S326046. PMID: 34557031; PMCID: PMC8453643.

[22] Zhou X, Pardue MT, Iuvone PM, Qu J. Dopamine signaling and myopia development: What are the key challenges. Prog Retin Eye Res. 2017;61:60-71. doi:10.1016/j.preteyeres.2017.06.003

[23] Jiang, X., Pardue, M. T., Mori, K., Ikeda, S. I., Torii, H., D'Souza, S., Lang, R. A., Kurihara, T., & Tsubota, K. (2021). Violet light suppresses lens-induced myopia via neuropsin (OPN5) in mice. Proceedings of the National Academy of Sciences of the United States of America, 118(22), e2018840118. https://doi.org/10.1073/pnas.2018840118

[24] Torii, H., Ohnuma, K., Kurihara, T., Tsubota, K., & Negishi, K. (2017). Violet Light Transmission is Related to Myopia Progression in Adult High Myopia. Scientific reports, 7(1), 14523. https://doi.org/10.1038/s41598-017-09388-7

[25] Zhang YY, Jiang WJ, Teng ZE, et al. Corneal curvature radius and associated factors in Chinese children: the Shandong Children Eye Study.PLoS One. 2015;10(2):e0117481. Published 2015 Feb 6. doi:10.1371/journal.pone.0117481

[26] Xiong S, Zhang B, Hong Y, He X, Zhu J, Zou H, Xu X. The Associations of Lens

For peer review only

For peer review only - http://bmjopen.bmj.com/site/about/guidelines.xhtml

Power With Age and Axial Length in Healthy Chinese Children and Adolescents Aged 6 to 18 Years. Invest Ophthalmol Vis Sci. 2017 Nov 1;58(13):5849-5855. doi: 10.1167/iovs.17-22639. PMID: 29141080.

[27] Wang Y, Liu Y, Zhu X, Zhou X, He JC, Qu X. Corneal and lenticular biometry in Chinese children with myopia. Clin Exp Optom. 2022 Aug 31:1-9. doi: 10.1080/08164622.2022.2116269. Epub ahead of print. PMID: 36045001.

[28] Metlapally R, Wildsoet CF. Scleral Mechanisms Underlying Ocular Growth and Myopia. Prog Mol Biol Transl Sci. 2015;134:241-8. doi: 10.1016/bs.pmbts.2015.05.005. Epub 2015 Jun 12. PMID: 26310158; PMCID: PMC4687967.

[29] Zhang D, Wang L, Jin L, Wen Y, Zhang X, Zhang L, Zhu H, Wang Z, Yu X, Xie C, Tong J, Shen Y. A Review of Intraocular Pressure (IOP) and Axial Myopia. J Ophthalmol. 2022 Jul 9;2022:5626479. doi: 10.1155/2022/5626479. PMID: 35855886; PMCID: PMC9288324.

[30] Wang P, Chen S, Liu Y, Lin F, Song Y, Li T, Aung T, Zhang X; GSHM study group. Lowering Intraocular Pressure: A Potential Approach for Controlling High Myopia Progression. Invest Ophthalmol Vis Sci. 2021 Nov 1;62(14):17. doi: 10.1167/iovs.62.14.17. PMID: 34787640; PMCID: PMC8606873.

[31] Hashemi H, Nabovati P, Khabazkhoob M, Emamian MH, Yekta A, Fotouhi A. Agreement of Central Corneal Thickness Measurements between Scheimpflug Photography and Optical Low-Coherence Reflectometry in Children. Semin Ophthalmol. 2020;35(4):252-256. doi:10.1080/08820538.2020.1810288

For peer review only - http://bmjopen.bmj.com/site/about/guidelines.xhtml

[32] Zheng Y, Huang G, Huang W, He M. Distribution of central and peripheral corneal thickness in Chinese children and adults: the Guangzhou twin eye study. Cornea. 2008;27(7):776-781. doi:10.1097/ICO.0b013e31816f62d3

[33] Zhou Q, Gao TY, Fan SJ, et al. Intraocular Pressure, Age, and Central Corneal Thickness in a Healthy Chinese Children Population: The Handan Offspring Myopia Study. Ophthalmic Epidemiol. 2022;29(5):499-506. doi:10.1080/09286586.2021.1966806

[34] Asharlous A, Hashemi H, Yekta A, et al. Topographic properties of the cornea in welders. Eur J Ophthalmol. 2022;32(1):165-169. doi:10.1177/1120672120974293

For peer review only

For peer review only - http://bmjopen.bmj.com/site/about/guidelines.xhtml

Table 1 Distribution of sample size and average altitude of each survey site

Groups		School	Sample Size (student)	Average altitude (meter)
**Tibet**	**Karo District**	Qamdo No. 1 Primary School	60	3250
		Qamdo Junior High School	30	
		Qamdo No. 1 Senior High School	120	
	**Drayab County**	Drayab County No. 1 Primary School	60	3170
		Drayab County Junior High School	30	
	**Tengchen County**	Xiexiong Township Central Primary School	60	3870
		Tengchen County Junior High School	30	
	**Markam County**	Qucainka Township Central Primary School	60	2300
		Mangkang County Junior High School	30	3865
	**Total**	/	480	/
**Chongqing**	**Shapingba District**	Fenghuang Experiental School	746	319
	**Banan District**	Banan Middle School	198	331
	**Total**	/	944	/

Table 2 Distribution of participants of different ages in Tibet and Chongqing groups

Age (year)	Tibet group			Chongqing group		
	Total	Males	Females	Total	Males	Females
**6**	22	10	12	29	15	14
**7**	38	17	21	59	24	35
**8**	36	20	16	69	39	30
**9**	32	20	12	55	22	33
**10**	39	19	20	53	34	19
**11**	42	19	23	78	39	39
**12**	53	34	19	86	40	46
**13**	49	18	31	79	35	44
**14**	44	25	19	74	49	25
**15**	38	20	18	66	35	31
**16**	28	11	17	61	13	48
**17**	19	10	9	37	9	28
**18**	8	3	5	2	2	0
**Total**	448	226	222	748	356	392

For peer review only

For peer review only - http://bmjopen.bmj.com/site/about/guidelines.xhtml

Table 3 Comparison of demographic data, myopia prevalence, and ocular biometric parameters in Tibetan and Chongqing children and adolescents

	Age (year)	Tibet group	Chongqing group	*p* value	Adjusted *p* value*
**Sex Males(%)**	Total	226/448 (50.4%)	356/748 (47.6%)	0.339	
**Age (year)**	Total	12.09 ± 3.15	12.20 ± 3.10	0.549	
**Myopia prevalence (%)**	6-8	11/96 (11.5%)	14/157 (8.9%)	0.511	
	9-11	39/113 (34.5%)	55/186 (29.6%)	0.372	
	12-14	69/146 (47.3%)	141/239 (59%)	0.025	
	15-18	59/93 (63.4%)	146/166 (88%)	< 0.001	
	Total	178/448 (39.7%)	356/748 (47.6%)	0.008	
**SE (D)**	6-8	-0.29 ± 1.37	-0.21 ± 1.15	0.629	
	9-11	-0.86 ± 1.61	-0.7 ± 1.12	0.347	
	12-14	-1.48 ± 1.84	-2.06 ± 2.08	0.006	
	15-18	-2.16 ± 1.90	-3.45 ± 1.90	< 0.001	
	Total	-1.21 ± 1.82	-1.65 ± 2.05	< 0.001	
**AL (mm)**	6-8	22.81 ± 0.76	22.94 ± 0.86	0.228	
	9-11	23.28 ± 0.85	23.57 ± 0.90	0.007	
	12-14	23.58 ± 1.01	24.17 ± 1.09	< 0.001	
	15-18	24.13 ± 1.03	24.86 ± 1.05	< 0.001	
	Total	23.45 ± 1.02	23.92 ± 1.19	< 0.001	
**Km (D)**	6-8	42.89 ± 1.49	43.24 ± 1.40	0.067	
	9-11	43.04 ± 1.34	43.17 ± 1.27	0.398	
	12-14	43.14 ± 1.34	43.30 ± 1.45	0.302	
	15-18	43.11 ± 1.49	43.32 ± 1.32	0.398	
	Total	43.06 ± 1.40	43.26 ± 1.36	0.014	
**AL/CR**	6-8	2.90 ± 0.08	2.94 ± 0.09	< 0.001	
	9-11	2.97 ± 0.10	3.01 ± 0.10	< 0.001	
	12-14	3.01 ± 0.12	3.10 ± 0.14	< 0.001	
	15-18	3.08 ± 0.13	3.19 ± 0.13	< 0.001	
	Total	2.99 ± 0.13	3.06 ± 0.15	< 0.001	
**CCT (μm)**	6-8	541.35 ± 32.45	545.72 ± 30.08	0.278	
	9-11	540.26 ± 27.32	552.67 ± 32.32	0.001	
	12-14	530.19 ± 30.83	551.84 ± 29.48	< 0.001	
	15-18	533.37 ± 28.51	548.43 ± 31.35	< 0.001	
	Total	535.78 ± 30.16	550.01 ± 30.80	< 0.001	
**WTW (mm)**	6-8	11.68 ± 0.40	11.75 ± 0.36	0.156	0.328
	9-11	11.65 ± 0.35	11.73 ± 0.35	0.071	0.771
	12-14	11.57 ± 0.39	11.70 ± 0.38	0.002	0.079
	15-18	11.63 ± 0.36	11.69 ± 0.39	0.224	0.437
	Total	11.62 ± 0.38	11.71 ± 0.37	< 0.001	0.039
**ACD (mm)**	6-8	3.01 ± 0.22	2.94 ± 0.26	0.031	0.003
	9-11	3.16 ± 0.25	3.06 ± 0.27	0.003	< 0.001
	12-14	3.18 ± 0.26	3.18 ± 0.27	0.775	0.001
	15-18	3.25 ± 0.26	3.24 ± 0.27	0.867	< 0.001
	Total	3.15 ± 0.26	3.11 ± 0.29	0.015	< 0.001
**IOP (mmHg)**	6-8	15.30 ± 2.64	16.52 ± 3.28	0.001	0.004
	9-11	14.94 ± 3.05	16.52 ± 2.88	< 0.001	0.001
	12-14	14.23 ± 3.20	16.61 ± 2.83	< 0.001	< 0.001
	15-18	14.01 ± 2.59	16.01 ± 2.87	< 0.001	< 0.001
	Total	14.59 ± 2.96	16.43 ± 2.95	< 0.001	< 0.001
**Astig. F (D)**	Total	1.17 ± 0.68	1.24 ± 0.68	0.086	
**Astig. B (D)**	Total	0.34 ± 0.14	0.36 ± 0.14	0.175	

For peer review only

For peer review only - http://bmjopen.bmj.com/site/about/guidelines.xhtml

* Adjusted AL(WTW, ACD) or CCT (IOP)

SE, spherical equivalent; D, diopter; AL, axial length; AL/CR: AL to corneal radius of curvature ratio; IOP, intraoccular pressure, D, diopter; Km, mean curvature power of the cornea within the central 3-mm circle; Astig. F, astigmatism of the front surface; Astig. B, astigmatism of the back surface; CCT, corneal thickness at the apex; WTW, white to white distance; ACD, internal anterior chamber depth.

Table 4 The results of multiple linear regression analysis between SE and AL and Km

Group		AL			Km			Adjusted R^2
		B	Sta.β	*P*	B	Sta.β	*P*	
**Tibet group**	**SE**	-1.567	-0.880	< 0.001	-0.649	-0.500	< 0.001	0.651
**Chongqing Group**	**SE**	-1.616	-0.940	< 0.001	-0.768	-0.510	< 0.001	0.777
**Total**	**SE**	-1.551	-0.905	< 0.001	-0.696	-0.486	< 0.001	0.728

B, Unstandardized Coefficient B, Sta.β, Standardized Coefficient Beta. AL, axial length; Km, mean curvature power of the cornea within the central 3-mm circle

Table 5 Comparison of ocular biometric parameters in altitude subgroups of primary school students

Parameters	Group A n = 398 Mean ± SD	Group B n = 58 Mean ± SD	Group C n = 121 Mean ± SD	Group D n = 60 Mean ± SD	*p*
**Sex Males(%)**	201 (50.5%)	31 (53.4%)	62 (51.2%)	31 (51.7%)	0.979
**Age (year)**	9.75 ± 1.85	9.87 ± 1.98	9.51 ± 2.03	9.83 ± 1.78	0.557
**Myopia prevalence**	99 (24.9%)	16 (27.6%)	39 (32.2%)	4 (6.7%)	0.002 (A,D: <0.05; B,D: <0.05; C,D: <0.05)
**SE (D)**	-0.64 ± 1.30	-0.64 ± 1.34	-0.80 ± 1.81	-0.15 ± 0.64	0.028 (C,D: 0.018)
**AL (mm)**	23.39 ± 0.98	23.19 ± 0.84	23.11 ± 0.90	22.97 ± 0.79	0.001 (A,C: 0.026; A,D: 0.007)
**IOP (mmHg)**	16.57 ± 3.05	16.12 ± 2.50	14.45 ± 3.05	15.17 ± 2.86	< 0.001 (A,C: <0.001; A,D: 0.004; B,C:0.004)
**AL/CR**	2.99 ± 0.11	2.94 ± 0.09	2.95 ± 0.11	2.92 ± 0.07	< 0.001 (A,B: 0.005; A,C: 0.001; A,D: <0.001)
**Km (D)**	43.22 ± 1.34	42.87 ± 1.29	43.11 ± 1.46	42.93 ± 1.59	0.189
**Astig. F (D)**	1.18 ± 0.64	1.11 ± 0.75	1.31 ± 0.74	1.05 ± 0.43	0.049
**Astig. B (D)**	0.33 ± 0.13	0.34 ± 0.14	0.35 ± 0.14	0.35 ± 0.11	0.492
**CCT (μm)**	550.11 ± 31.2	533.17 ± 30.12	545.77 ± 28.13	532.4 ± 32.50	< 0.001 (A,B: 0.001; A,D: <0.001; C,D: 0.036)
**WTW (mm)**	11.73 ± 0.36	11.63 ± 0.36	11.63 ± 0.38	11.71 ± 0.42	0.024 (A,C: 0.048)
**ACD (mm)**	3.03 ± 0.27	3.16 ± 0.27	3.08 ± 0.24	3.07 ± 0.25	0.004 (A,B: 0.004)

SE, spherical equivalent; D, diopter; AL, axial length; AL/CR: AL to corneal radius of curvature ratio; IOP, intraoccular pressure, D, diopter; Km, mean curvature power of the cornea; Astig. F, astigmatism of the front surface; Astig. B, astigmatism of the back surface; CCT, corneal thickness at the apex; WTW, white to white distance; ACD, internal anterior chamber depth.

For peer review only

For peer review only - http://bmjopen.bmj.com/site/about/guidelines.xhtml

For peer review only

For peer review only - http://bmjopen.bmj.com/site/about/guidelines.xhtml

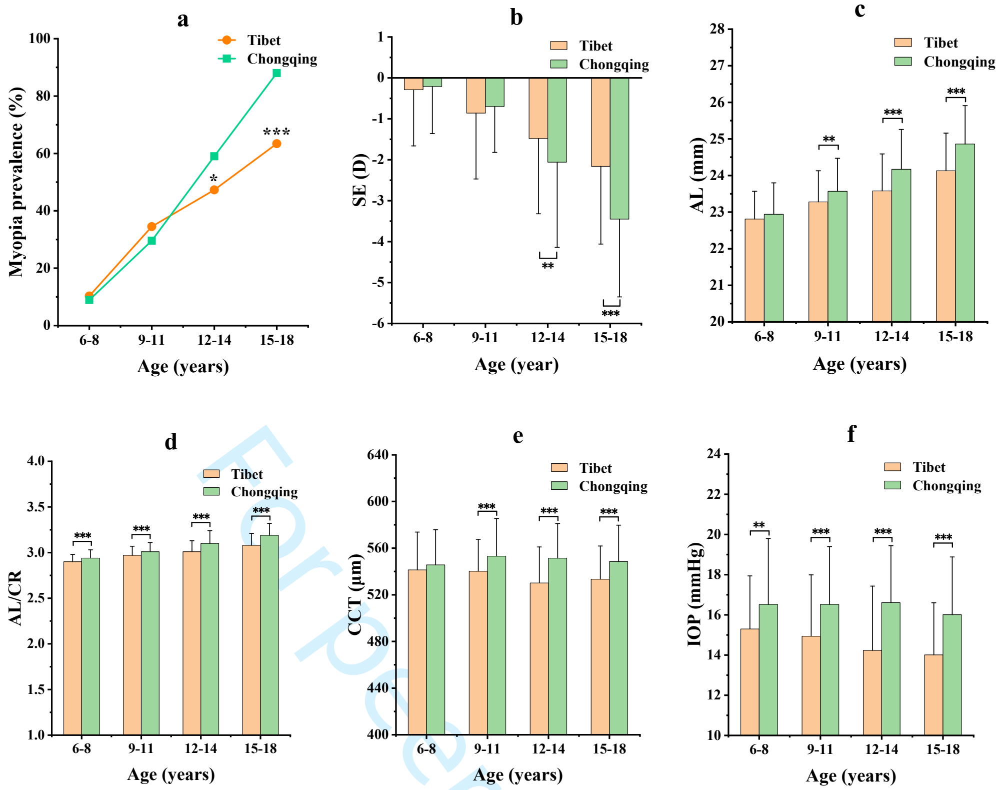

Figure 1 Comparison of SE, AL, CCT and IOP in Tibetan and Chongqing children and adolescents by age groups. SE, spherical equivalent; D, diopter; AL, axial length; AL/CR, AL to corneal radius of curvature ratio; CCT, corneal thickness at the apex;IOP, intraoccular pressure

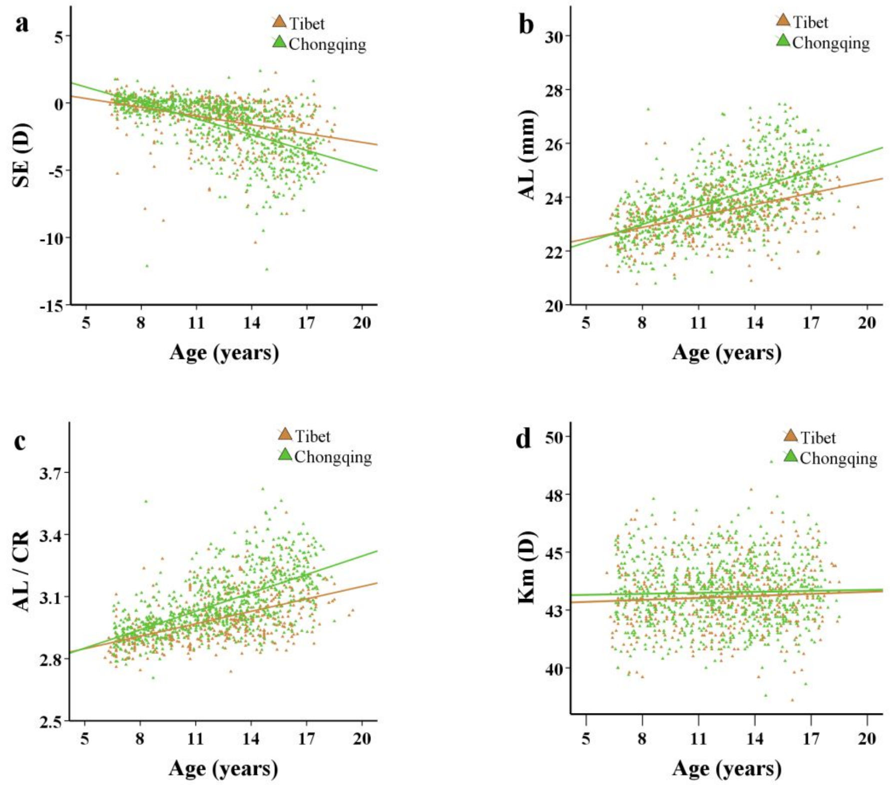

For peer review only - http://bmjopen.bmj.com/site/about/guidelines.xhtml

Figure 2 Linear regression model of SE, AL, AL/CR and Km with age.
SE, spherical equivalent; D, diopter; AL, axial length; AL/CR, AL to corneal radius of curvature ratio; Km, mean curvature power of the cornea

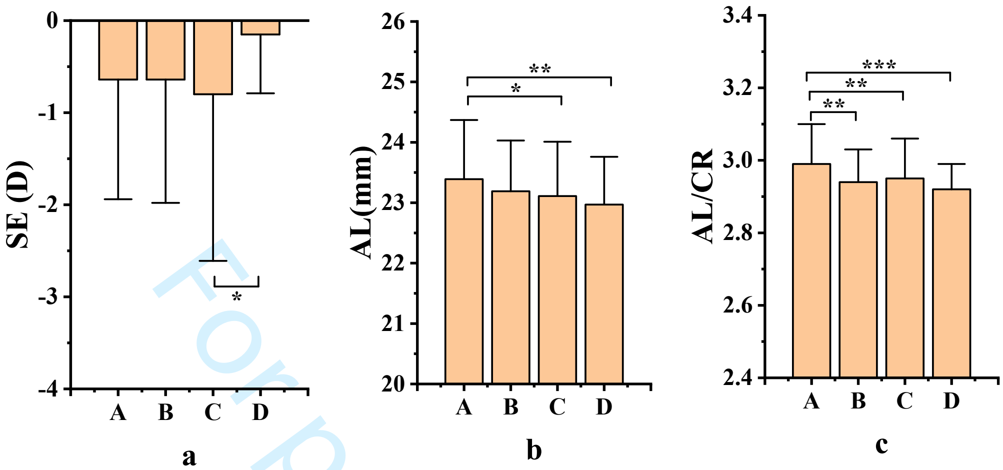

Figure 3 Comparison of SE, AL and AL/CR in altitude subgroups of primary school students. SE, spherical equivalent; D, diopter; AL, axial length; AL/CR, AL to corneal radius of curvature ratio. Group A, with an average altitude of 325 meters; Group B, with an average altitude of 2,300 meters; Group C, with an average altitude of 3,250 and 3,170 meters, Group D, with an average altitude of 3,870 meters.

For peer review only - http://bmjopen.bmj.com/site/about/guidelines.xhtml

STROBE Statement—Checklist of items that should be included in reports of ***cross-sectional studies***

	Item No	Recommendation	Page No
**Title and abstract**	1	(*a*) Indicate the study's design with a commonly used term in the title or the abstract	1
		(*b*) Provide in the abstract an informative and balanced summary of what was done and what was found	1
**Introduction**			
Background/rationale	2	Explain the scientific background and rationale for the investigation being reported	3
Objectives	3	State specific objectives, including any prespecified hypotheses	5
**Methods**			
Study design	4	Present key elements of study design early in the paper	5
Setting	5	Describe the setting, locations, and relevant dates, including periods of recruitment, exposure, follow-up, and data collection	5
Participants	6	(*a*) Give the eligibility criteria, and the sources and methods of selection of participants	5
Variables	7	Clearly define all outcomes, exposures, predictors, potential confounders, and effect modifiers. Give diagnostic criteria, if applicable	6
Data sources/ measurement	8*	For each variable of interest, give sources of data and details of methods of assessment (measurement). Describe comparability of assessment methods if there is more than one group	
Bias	9	Describe any efforts to address potential sources of bias	6
Study size	10	Explain how the study size was arrived at	6
Quantitative variables	11	Explain how quantitative variables were handled in the analyses. If applicable, describe which groupings were chosen and why	6
Statistical methods	12	(*a*) Describe all statistical methods, including those used to control for confounding	7
		(*b*) Describe any methods used to examine subgroups and interactions	7
		(*c*) Explain how missing data were addressed	7
		(*d*) If applicable, describe analytical methods taking account of sampling strategy	7
		(*e*) Describe any sensitivity analyses	
**Results**			
Participants	13*	(a) Report numbers of individuals at each stage of study—eg numbers potentially eligible, examined for eligibility, confirmed eligible, included in the study, completing follow-up, and analysed	7
		(b) Give reasons for non-participation at each stage	
		(c) Consider use of a flow diagram	
7Descriptive data	14*	(a) Give characteristics of study participants (eg demographic, clinical, social) and information on exposures and potential confounders	
		(b) Indicate number of participants with missing data for each variable of interest	
Outcome data	15*	Report numbers of outcome events or summary measures	
Main results	16	(*a*) Give unadjusted estimates and, if applicable, confounder-adjusted estimates and their precision (eg, 95% confidence interval). Make clear which confounders were adjusted for and why they were included	8

For peer review only - http://bmjopen.bmj.com/site/about/guidelines.xhtml

		(*b*) Report category boundaries when continuous variables were categorized	
		(*c*) If relevant, consider translating estimates of relative risk into absolute risk for a meaningful time period	
Other analyses	17	Report other analyses done—eg analyses of subgroups and interactions, and sensitivity analyses	9
**Discussion**			
Key results	18	Summarise key results with reference to study objectives	10
Limitations	19	Discuss limitations of the study, taking into account sources of potential bias or imprecision. Discuss both direction and magnitude of any potential bias	14
Interpretation	20	Give a cautious overall interpretation of results considering objectives, limitations, multiplicity of analyses, results from similar studies, and other relevant evidence	14
Generalisability	21	Discuss the generalisability (external validity) of the study results	14
**Other information**			
Funding	22	Give the source of funding and the role of the funders for the present study and, if applicable, for the original study on which the present article is based	15

*Give information separately for exposed and unexposed groups.

Note: An Explanation and Elaboration article discusses each checklist item and gives methodological background and published examples of transparent reporting. The STROBE checklist is best used in conjunction with this article (freely available on the Web sites of PLoS Medicine at http://www.plosmedicine.org/, Annals of Internal Medicine at http://www.annals.org/, and Epidemiology at http://www.epidem.com/). Information on the STROBE Initiative is available at www.strobe-statement.org.

For peer review only

Myopia Prevalence and Ocular Biometry in Children and Adolescents at Different Altitudes: A Cross-sectional Study in Chongqing and Tibet, China

Journal:	*BMJ Open*
Manuscript ID	bmjopen-2023-078018.R1
Article Type:	Original research
Date Submitted by the Author:	10-Jan-2024
Complete List of Authors:	Xiang, Yongguo; The First Affiliated Hospital of Chongqing Medical University; Chongqing Medical University<br>Cheng, Hong; The First Affiliated Hospital of Chongqing Medical University; Chongqing Medical University<br>Sun, Kexin; The First Affiliated Hospital of Chongqing Medical University; Chongqing Medical University<br>Zheng, Shijie; The First Affiliated Hospital of Chongqing Medical University; Chongqing Medical University<br>Du, Miaomiao; The First Affiliated Hospital of Chongqing Medical University; Chongqing Medical University<br>Gao, Ning; The First Affiliated Hospital of Chongqing Medical University; Chongqing Medical University<br>Zhang, Tong; Chongqing Medical University<br>Yang, Xin; The First Affiliated Hospital of Chongqing Medical University<br>Xia, Jiuyi; The First Affiliated Hospital of Chongqing Medical University<br>Huang, Rongxi; Chongqing General Hospital<br>Wan, Wenjuan; The First Affiliated Hospital of Chongqing Medical University; Chongqing Medical University<br>Hu, Ke; The First Affiliated Hospital of Chongqing Medical University; Chongqing Medical University
**Primary Subject Heading**:	Ophthalmology
Secondary Subject Heading:	Ophthalmology
Keywords:	EPIDEMIOLOGIC STUDIES, Paediatric ophthalmology < OPHTHALMOLOGY, Medical ophthalmology < OPHTHALMOLOGY, Corneal and external diseases < OPHTHALMOLOGY

SCHOLARONE™
Manuscripts

I, the Submitting Author has the right to grant and does grant on behalf of all authors of the Work (as defined in the below author licence), an exclusive licence and/or a non-exclusive licence for contributions from authors who are: i) UK Crown employees; ii) where BMJ has agreed a CC-BY licence shall apply, and/or iii) in accordance with the terms applicable for US Federal Government officers or employees acting as part of their official duties; on a worldwide, perpetual, irrevocable, royalty-free basis to BMJ Publishing Group Ltd ("BMJ") its licensees and where the relevant Journal is co-owned by BMJ to the co-owners of the Journal, to publish the Work in this journal and any other BMJ products and to exploit all rights, as set out in our licence.

The Submitting Author accepts and understands that any supply made under these terms is made by BMJ to the Submitting Author unless you are acting as an employee on behalf of your employer or a postgraduate student of an affiliated institution which is paying any applicable article publishing charge ("APC") for Open Access articles. Where the Submitting Author wishes to make the Work available on an Open Access basis (and intends to pay the relevant APC), the terms of reuse of such Open Access shall be governed by a Creative Commons licence – details of these licences and which Creative Commons licence will apply to this Work are set out in our licence referred to above.

Other than as permitted in any relevant BMJ Author's Self Archiving Policies, I confirm this Work has not been accepted for publication elsewhere, is not being considered for publication elsewhere and does not duplicate material already published. I confirm all authors consent to publication of this Work and authorise the granting of this licence.

For peer review only

Myopia Prevalence and Ocular Biometry in Children and Adolescents at Different Altitudes: A Cross-sectional Study in Chongqing and Tibet, China

Yongguo Xiang[1,2]*, Hong Cheng[1,2]*, Kexin Sun[1,2], Shijie Zheng[1,2], Miaomiao Du[1,2], Ning Gao[1,2], Tong Zhang[2], Xin Yang[1], Jiuyi Xia[1], Rongxi Huang[3], Wenjuan Wan[1,2#] and Ke Hu[1,2#]

1 The First Affiliated Hospital of Chongqing Medical University, Chongqing Key Laboratory of Ophthalmology, Chongqing Eye Institute, Chongqing Branch (Municipality Division) of National Clinical Research Center for Ocular Diseases, Chongqing, People's Republic of China

2 Chongqing Medical University, Chongqing, People's Republic of China

3 Chongqing General Hospitoal, Chongqing, People's Republic of China

*These authors contributed equally to this work

[#]Corresponding Author: Wenjuan Wan, wanwenjuancqums@163.com, and Ke Hu, 42222@qq.com

Objective: To investigate the differences in myopia prevalence and ocular biometry in children and adolescents in Chongqing and Tibet, China.

Design: Cross-sectional study.

Setting: The study included children and adolescents aged 6-18 years in Chongqing, a low-altitude region, and in Qamdo, a high-altitude region of Tibet.

Participants: A total of 448 participants in Qamdo, Tibet, and 748 participants in Chongqing were enrolled in this study.

Methods: All participants underwent uncorrected visual acuity (UCVA) assessment, non-cycloplegic refraction, axial length (AL) measurement, intraocular pressure (IOP) measurement, and corneal tomography. And the participants were grouped according

For peer review only - http://bmjopen.bmj.com/site/about/guidelines.xhtml

to age (6-8, 9-11, 12-14, and 15-18 years group), and altitude of location [primary school students: Group A (average altitude: 325 meters), Group B (average altitude: 2,300 meters), Group C (average altitude: 3,250 and 3,170 meters), Group D (average altitude: 3,870 meters)].

Results: There was no statistical difference in mean age (12.09 ± 3.15 vs. 12.2 ± 3.10, $p = 0.549$) and sex distribution (males, 50.4% vs. 47.6%, $p = 0.339$) between the two groups. The Tibet group presented greater spherical equivalent [SE, -0.63 (-2.00, 0.13) vs.-0.88 (-2.88, -0.13), $p < 0.001$], shorter AL (23.45 ± 1.02 vs. 23.92 ± 1.19, $p < 0.001$), lower prevalence of myopia (39.7% vs. 47.6%, $p = 0.008$), and flatter mean curvature power of the cornea (Km, 43.06 ± 1.4 vs. 43.26 ± 1.36, $p = 0.014$) than the Chongqing group. Further analysis based on age subgroups revealed that the Tibet group had a lower prevalence of myopia and higher SE in the 12-14, and 15-18 years old groups, shorter AL in the 9-11, 12-14, and 15-18 years old groups, and lower AL to corneal radius of curvature ratio (AL/CR) in all age subgroups compared to the Chongqing group, while Km was similar between the two groups in each age subgroup. Simple linear regression analysis showed that SE decreased with age in both the Tibet and Chongqing groups, with the Tibet group exhibiting a slower rate of decrease ($p < 0.001$). AL and AL/CR increased with age in both the Tibet and Chongqing groups, but the rate of increase was slower in the Tibet group ($p < 0.001$ of both). Multiple linear regression analysis revealed that AL had the greatest effect on SE in both groups, followed by Km. In addition, the children and adolescents in Tibet presented thinner corneal thickness (CCT, $p < 0.001$), smaller white to white

For peer review only - http://bmjopen.bmj.com/site/about/guidelines.xhtml

distance (WTW, $p < 0.001$), lower IOP ($p < 0.001$), and deeper anterior chamber depth (ACD, $p = 0.015$) than in Chongqing. Comparison of altitude subgroups showed that the prevalence of myopia ($p = 0.002$), SE ($p = 0.031$), AL ($p = 0.001$), and AL/CR ($p < 0.001$) of children at different altitudes were statistically different but the Km ($p = 0.189$) were similar. The highest altitude, Tengchen County, exhibited the lowest prevalence of myopia and greatest SE among children, and the mean AL also decreased with increasing altitude.

Conclusions: Myopia prevalence in Tibet was comparable to that in Chongqing for students aged 6-8 and 9-11 years but was lower and myopia progressed more slowly for students aged 12-14 and 15-18 years than in Chongqing, and AL was the main contributor for this difference, which may be related to higher ultraviolet radiation exposure and lower IOP in children and adolescents at high altitude in Tibet. Differences in AL and AL/CR between Tibet and Chongqing children and adolescents manifested earlier than in SE, underscoring the importance of AL measurement in myopia screening.

Strengths and limitations of this study:

- This study investigated the differences in myopia prevalence and ocular biometry in children and adolescents residing at different altitudes in China.
- The limited sample size employed in this study may hinder a comprehensive evaluation of the ocular biometric characteristics in children and adolescents.

For peer review only - http://bmjopen.bmj.com/site/about/guidelines.xhtml

- The vigorous accommodative system in children may introduce potential inaccuracies in noncycloplegic refraction, potentially resulting in an overestimation of myopia.

BACKGROUND

It is reported that approximately 400 million people worldwide live at altitudes above 1,500 meters[1]. The Qinghai-Tibet Plateau is the highest geographical unit in the world. Qamdo is located in the eastern part of the Qinghai-Tibet Plateau, with an average altitude of more than 3,500 meters. It is characterized by low atmospheric pressure, hypoxia, cold and dry weather, and strong ultraviolet radiation (UVR)[2]. Previous study have shown that UVR intensity increases by 10-12% for every 1000 meters of altitude. And approximately 90-99% of the solar UVR energy that reaches the earth's surface is UVA (315-400 nm wavelength), whereas only 1-10% is UVB (280-315nm wavelength)[3]. The cornea sits at the anterior aspect of the eye and, like the skin, is highly exposed to UVR. Torii et al.[4] found that violet light (VL, 360-400 nm wavelength) suppresses myopia progression by upregulating the myopia suppressive gene EGR1. Kobashi et al.[5] reported that there is a small amount of riboflavin in the human corneal stroma, and the ex vivo corneal stiffness significantly increases after VL (375 nm wavelength) irradiation based on physiological riboflavin. Epidemiological data show that the prevalence of keratoconus, pterygium, dry eye disease, cataract, and strabismus is significantly higher in the highlands than in the lowlands[2][6][7][8][9], and the cornea of the highlanders is thinner than that of the lowlanders[10][11]. Our previous study also found that the prevalence of myopia

For peer review only - http://bmjopen.bmj.com/site/about/guidelines.xhtml

among children and adolescents in Tibet was lower than that in the plains[12]. However, it is unclear whether ocular biometry is associated with myopia prevalence between high and low altitude populations with different levels of UVR exposure.

The axial length (AL) and AL to corneal radius of curvature ratio (AL/CR) have been reported to be strongly correlated with ocular refraction, and play an important role in the screening and monitoring of myopia[13]. Cycloplegic refraction is considered the gold standard for measuring refractive errors, but limited by the fact that mydriatic drops can induce mydriasis and cycloplegia[14] Consequently, noncycloplegic refraction remains a dependable evaluation index in large scale myopia investigations.

In this study, we compared the prevalence of myopia and ocular biometry in children and adolescents in Chongqing and Qamdo, Tibet, and attempted to explore the effect of altitude on ocular biometry.

MATERIALS AND METHODS

This school-based cross-sectional study was conducted among children and adolescents aged 6-18 years in Chongqing, a low-altitude region, and in Qamdo, a high-altitude region of Tibet. The Ethics Committee of the First Affiliated Hospital of Chongqing Medical University approved this study and it was conducted in accordance with the tenets of the Declaration of Helsinki. At least one parent or legal guardian of each participant was informed about the study and signed an informed consent form.

Study Population

This study selected Karo district, Tengchen county, Markam county, and Drayab

For peer review only

For peer review only - http://bmjopen.bmj.com/site/about/guidelines.xhtml

county in Qamdo, Tibet, as well as Shapingba district and Banan district in Chongqing, as the investigation sites. A stratified sampling method was employed to select students ranging from the first grade through the senior second level at each investigation site. Ultimately, a total of 480 students from Qamdo, Tibet, and 944 students from Chongqing were included in this school-based study. The specifics of the sample inclusion are presented in Table 1.

Methods

All participants underwent a standardized ophthalmic examination. Uncorrected visual acuity (UCVA) was assessed and recorded in LogMAR scores by using the standard logarithmic visual acuity chart at 5 meters. Noncycloplegic refraction was performed with an autorefractor (Supore, China). Axial length (AL) was measured with the AL-scan (NIDEK, Japan). Intraocular pressure (IOP) was assessed with a non-contact tonometer (NIDEK, Japan). Corneal and anterior chamber parameters were measured with the Pentacam AXL (Oculus Optikgeräte GmbH, Wetzlar, Germany). The quality specification section on the output graph was used to assess the quality, with an "OK" reading indicating an acceptable quality. All examinations were performed by skilled ophthalmologists. At least three measurements were made for each eye and averaged, but only one eye of each participant was randomly selected for statistical analysis. Spherical equivalent (SE) equals diopter (D) of spherical power plus 1/2 diopter (D) of cylindrical power. Myopia was defined as SE <-0.5D and UCVA >0 logMAR.

The investigated indices were as follows: UCVA, SE, AL, IOP, AL to corneal radius

For peer review only

For peer review only - http://bmjopen.bmj.com/site/about/guidelines.xhtml

of curvature ratio (AL/CR); mean curvature power of the cornea within the central 3-mm circle (Km), astigmatism of the front surface (Astig. F); astigmatism of the back surface (Astig. B); corneal thickness at the apex (CCT); white to white distance (WTW); internal anterior chamber depth (ACD), and ACD/AL.

The participants were grouped according to age (6-8 years group, 9-11 years group, 12-14 years group, and 15-18 years group), and altitude of location [primary school students: Group A (Shapingba and Banan district, average altitude: 325 meters), Group B (Qucainka township, average altitude: 2,300 meters), Group C (Karo district and Drayab county, average altitude: 3,250 and 3,170 meters, respectively), Group D (Xiexiong Township, average altitude: 3,870 meters)].

Patient and public involvement statement

Patients or the public were not involved in the design, or conduct, or reporting, or dissemination plans of our research

Statistical Analysis

The statistical analysis was performed with SPSS version 26 (IBM Corp. in Armonk, NY, USA). One-sample Kolmogorov–Smirnov tests and the normal distribution histogram method were used to assess the normality of the distributions of the continuous variables. Independent samples t-test and analysis of variance (ANOVA) were employed to compare normally and approximately normally distributed continuous variables, and Mann-Whitney U test and Kruskal-Wallis H test were used to compare heavily skewed continuous variables. Dunn-Bonferroni post-hoc test was conducted for pairwise comparisons. ANCOVA test was applied to compare IOP,

For peer review only - http://bmjopen.bmj.com/site/about/guidelines.xhtml

156 WTW, and ACD between the Tibet and Chongqing groups, with adjustments made for CCT or AL. The chi-squared test was utilized to compare categorical variables. Simple linear regression analysis was performed to examine the relationship between SE, AL, AL/CR, and age. Multiple linear regression analysis was conducted to explore the relationship between AL, Km, and SE. The significance level was set at $p < 0.05$.

162 ***RESULTS***

163 Thirty-two students from Tibet and 181 students from Chongqing were excluded due to lack of qualified ocular biometric results, and 15 students from Chongqing were excluded for wearing corneal contact lenses. Finally, a total of 448 students in Tibet and 748 students in Chongqing were enrolled in this study. The distribution of participants by gender and age is presented in Table 2. And there were no statistical difference observed in the mean age (12.09 ± 3.15 vs. 12.2 ± 3.10, $p = 0.549$, Table 3) and sex distribution (males, 50.4% vs. 47.6%, $p = 0.339$, Table 3) between the two groups.

171 **Comparison between the Tibet group and the Chongqing group**

172 As shown in Table 3, the Tibet group presented a lower prevalence of myopia (39.7% vs. 47.6%, $p = 0.008$), greater SE [-0.63 (-2.00, 0.13) vs.-0.88 (-2.88, -0.13), p < 0.001], shorter AL (23.45 ± 1.02 vs. 23.92 ± 1.19, $p < 0.001$), flatter Km (43.06 ± 1.4 vs. 43.26 ± 1.36, $p = 0.014$), smaller anterior corneal surface astigmatism[1.05 (0.70, 1.40) vs. 1.10 (0.80, 1.60), $p = 0.036$], and smaller AL/CR (2.99 ± 0.13 vs.3.06 ± 0.15, $p < 0.001$) than the Chongqing group, while the astigmatism of the posterior

For peer review only - http://bmjopen.bmj.com/site/about/guidelines.xhtml

corneal surface was similar between the two groups [0.30 (0.30, 0.40) vs. 0.30 (0.30, 0.40), p = 0.326]. Further analysis based on age subgroups revealed that the Tibet group had a lower prevalence of myopia and higher SE in the 12-14, and 15-18 years old groups, shorter AL in the 9-11, 12-14, and 15-18 years old groups, and lower AL/CR in all age subgroups compared to the Chongqing group, while Km was similar between the two groups in each age subgroup (Table 3, Supplemental Figure S1a, S1b, S1c, S1d and S1e).

The children and adolescents in Tibet exhibited thinner CCT (535.78 ± 30.16 vs. 550.01 ± 30.8, $p < 0.001$), smaller WTW (11.62 ± 0.38 vs. 11.71 ± 0.37, $p < 0.001$, after adjustment for AL, p = 0. 039), deeper ACD (3.15 ± 0.26 vs. 3.11 ± 0.29, p = 0.015, after adjustment for AL, $p < 0.001$), lower IOP (14.59 ± 2.96 vs. 16.43 ± 2.95, $p < 0.001$, after adjustment for CCT, $p < 0.001$), and greater ACD/AL (0.134 ± 0.009 vs. 0.130 ± 0.010, $p < 0.001$) than those in Chongqing. Subgroup analysis based on age revealed similar differences in CCT, IOP, and ACD/AL (Table 3, Supplemental Figure S1f, S1g, and S1h). Furthermore, after adjusting for AL, the ACD was found to be deeper in the Tibet group across all age subgroups, while the WTW remained similar.

Regression analysis

The simple linear regression analysis showed that SE decreased with age in both the Tibet ($R^2 = 0.140$, $p < 0.001$) and Chongqing ($R^2 = 0.352$, $p < 0.001$) groups, with the Tibet group exhibiting a slower rate of decrease (B: -0.217 vs. -0.393, $p < 0.001$, Supplemental Figure S2a). Additionally, AL and AL/CR increased with age in both

For peer review only - http://bmjopen.bmj.com/site/about/guidelines.xhtml

Tibet (AL: R2 = 0.192, $p < 0.001$; AL/CR: $R^2 = 0.253$, $p < 0.001$) and Chongqing (AL: $R^2 = 0.335$, $p < 0.001$; AL/CR: $R^2 = 0.391$, $p < 0.001$) groups, but the rate of increase was slower in the Tibet group (B: AL: 0.143 vs. 0.223, $p < 0.001$, Figure S2b; AL/CR: 0.02 vs. 0.03, $p < 0.001$, Figure S2c). Conversely, there was no linear relationship between Km and age for both the Tibet ($R^2 = 0.004$, $p = 0.173$) and Chongqing ($R^2 = 0.001$, $p = 0.377$, Figure S2d) groups. Furthermore, the multiple linear regression analysis revealed that AL had the greatest effect on SE in both groups, followed by Km (Table 4).

Comparison of children among altitude subgroups

As shown in Table 5, there was no statistical difference in the mean age ($p = 0.557$) and sex distribution ($p = 0.979$) among the altitude subgroups. Comparison of altitude subgroups showed that the prevalence of myopia ($p = 0.002$), SE ($p = 0.031$, Figure S3a), AL ($p = 0.001$, Figure S3b), AL/CR ($p < 0.001$, Figure S3c), and ACD/AL ($p < 0.001$, Figure S3d) of children at different altitudes were statistically different but the Km ($p = 0.189$) were similar. Notably, the highest altitude, Tengchen County, exhibited the lowest prevalence of myopia among children, and the mean AL value also decreased with increasing altitude.

DISCUSSION

In this school-based cross-sectional study, we conducted a comparison of the prevalence of myopia and ocular biometry among students aged 6-18 years in Chongqing and Qamdo, Tibet. Our findings indicate that the prevalence of myopia and SE were comparable between the two locations during the age groups of 6-8 years

For peer review only - http://bmjopen.bmj.com/site/about/guidelines.xhtml

and 9-11 years. However, we observed a lower prevalence of myopia and a higher SE in Tibet compared to Chongqing among students aged 12-14 years and 15-18 years. This discrepancy may be attributed to the slower progression of myopia with advancing age in the Tibetan population. The Tibetan students exhibited a shorter AL within the 9-11, 12-14, and 15-18 years old age groups, as well as lower AL/CR across all age subgroups in comparison to the students from Chongqing. However, the Km values were found to be similar between the two locations within each respective age subgroup.

Myopia is one of the most common eye disorders globally, caused by both environmental and genetic risk factors[15]. And the increasing prevalence of myopia can be largely explained by increased educational pressures and reductions in the amount of time that children spend outdoors[16]. Qian et al.[17] and Wang et al.[12] have reported that Tibetan children and adolescents at high altitudes had a lower prevalence of myopia than their counterparts at low altitudes. In line with their findings, our study also revealed that children and adolescents in Tibet presented a lower overall prevalence of myopia, shorter AL, higher SE, and slower myopia progression in comparison to individuals in Chongqing. Furthermore, the prevalence of myopia among children and adolescents in Tibet was found to be lower than previous reports in other plain regions of China[18][19][20]. Interestingly, the prevalence of myopia and SE of children aged 6-8 and 9-11 years in Tibet in our study were similar to those in Chongqing, and similar findings were reported by Wu et al[21], suggesting that myopia is widespread among children in high altitude

For peer review only - http://bmjopen.bmj.com/site/about/guidelines.xhtml

regions and that myopia prevention and control in high altitude areas of China are equally challenging.

In recent years, much research evidence suggests that outdoor activity in bright light conditions is a protective factor against myopia[15][16]. The mechanism for this protective effect appears to be related to an increased release of dopamine stimulated by visible light (400-700 nm wavelength), which inhibits increased axial elongation[22]. In addition, Tsubota's team reported that VL, with wavelengths from 360 to 400 nm, can effectively suppress myopia progression in chicks, mice, and humans[4][23]**Error! Reference source not found.**. Qamdo is located in the eastern part of the Qinghai-Tibet Plateau, with an average altitude of more than 3,500 meters, and the UVR intensity, especially UVA intensity (315-400 nm wavelength), is stronger in Qamdo than that in Chongqing due to the increase in UVR intensity with altitude[3]. Additionally, Qamdo experiences an average daily sunshine duration of 7.18 hours, approximately twice the duration observed in Chongqing, which amounts to 3.54 hours https://www.weather2visit.com/). The results of our study indicated that children and adolescents in Tibet exhibited shorter AL and slower AL elongation in comparison to their counterparts in Chongqing. Furthermore, our analysis of altitude subgroups revealed a decrease in the mean value of AL with increasing altitude. Although information regarding outdoor activities was not collected in this study for both groups, our previous research demonstrated that children and adolescents in Tibet spend more time outdoors than those in Chongqing[12], which

For peer review only - http://bmjopen.bmj.com/site/about/guidelines.xhtml

may be synergistic with stronger UVR intensity and longer average daily sunshine in Tibet in slowing myopia progression.

The findings from the multiple linear regression analysis indicated that the AL and Km were the primary determinants of SE. Notably, the Km remained consistent with age in both Tibet and Chongqing children and adolescents. Consequently, the AL emerged as the primary factor contributing to the disparity in myopia progression between the two locations. Moreover, the differences in AL between Tibet and Chongqing children and adolescents manifested earlier than those in SE, underscoring the importance of AL measurement in myopia screening. Corneal curvature as well as ACD also contribute to myopic progression[25]. In the case of children and adolescents in Tibet, the mean corneal curvature was found to be flatter compared to those in Chongqing, albeit with a minimal impact on the difference in myopia. However, after adjusting for AL, it was observed that the ACD in Tibetan children and adolescents was deeper than that in Chongqing. This suggested that the lens in Tibetan individuals may be either flatter or positioned more posteriorly, resulting in a stronger compensatory effect on the elongation of AL[26][27]. However, regrettably, the absence of lens curvature data is attributed to the constraints of the examination equipment, and the investigation into the association between lens curvature and myopia will be undertaken in subsequent research.

The mechanism of myopia development was considered to be associated with scleral matrix remodeling[28]. And scleral matrix remodeling has been shown to contribute to the biomechanical susceptibility of the sclera to accommodation-induced IOP

For peer review only - http://bmjopen.bmj.com/site/about/guidelines.xhtml

fluctuations, resulting in reduced scleral thickness, AL elongation, and axial myopia. The rise in IOP can increase the burden of scleral stretching and cause axial lengthening[29]. In addition, lowering IOP was also considered a potential approach for controlling the progression of high myopia[30]. Several clinical studies have examined the association between IOP and the progression of myopia, but the results were inconsistent[29]. In our study, we observed that IOP levels were consistently higher in children and adolescents residing in Chongqing compared to those in Tibet, across all age subgroups. In a cross-sectional, multicenter, population-based study including 284,937 participants conducted by Liu et al.[31]**Error! Reference source not found.**, they also reported that IOP was significantly higher in low-altitude populations than in high-altitude populations. This disparity persisted after adjusting for CCT, which may suggest a potential link between elevated IOP and myopia. However, further longitudinal studies are required to establish a causal relationship between IOP and AL elongation. The underlying reasons for the observed disparity in IOP between these two populations remain unclear.

In our study, it was observed that Tibetan children and adolescents exhibited a thinner corneal thickness compared to their counterparts in Chongqing. This finding aligns with previous research conducted by Amit et al. [10] and Patyal et al. [11], who also reported a thinner CCT in individuals residing at higher altitudes. Additionally, our study revealed that the CCT of Tibetan children and adolescents was thinner than what was previously observed in studies conducted at lower altitudes[32][33][34]. This discrepancy may be attributed to variations in UVR exposure levels, as

For peer review only - http://bmjopen.bmj.com/site/about/guidelines.xhtml

evidenced by the study conducted by Asharlous et al.[35], which found that welders, who are exposed to long-term UVR, exhibited a significantly thinner cornea compared to healthy controls. Hence, it is plausible to consider long-term UVR exposure as a potential contributor to corneal thinning.

Our current study had several limitations. Firstly, the limited sample size employed in this study may hinder a comprehensive evaluation of the ocular biometric characteristics in children and adolescents residing in the plateau region. Secondly, the accommodative system of children is vigorous, which may introduce inaccuracies in noncycloplegic refraction, potentially resulting in an overestimation of myopia. Lastly, this study primarily examined variations in ocular biometry among children and adolescents living at different altitudes. However, further research is needed to explore the potential correlation between ocular biometry and behavioral habits. In addition, cross-sectional studies cannot accurately assess the progression of myopia. In the future, we will conduct prospective studies to provide more reliable conclusions.

In conclusion, this study investigated the prevalence of myopia and ocular biometry of children and adolescents aged 6-18 years in Chongqing and Qamdo, Tibet. Students aged 12-18 years had a lower prevalence of myopia and greater SE in Tibet than in Chongqing, but no discernible difference was observed among students aged 6-11 years, indicating a comparatively slower progression of myopia in Tibetan students, which may be related to higher UVR exposure, flatter corneas, and lower IOP in Tibetan children and adolescents. In addition, we found that the Tibetan

For peer review only - http://bmjopen.bmj.com/site/about/guidelines.xhtml

population had thinner corneas than the Chongqing population, which may be associated with long-term UVR exposure.

Funding

This work was supported by the National Natural Science Foundation of China (Grant nos. 81870650, 82000883, 81900885 and 81970832), the Project Foundation of Chongqing Science and Technology Commission of China (Grant nos. CSTC2021jscx-gksb-N0017, cstc2020jcyj-msxmX0829, CSTB2022NSCQ-MSX1561, and cstc2021jcyj-msxmX0967) and Chongqing Talent Plan "Contract program" (Grant no. cstc2022ycjh-bgzxm0121).

Competing interests

The authors declare no competing interests.

Author contributions

Study conception and design: YGX, HC, WJW and KH; data collection and statistics: YGX, HC, KXS, SJZ, MMD, NG, TZ, XY, JYX and RXH; data interpretation: YGX and HC; writing-original draft: YGX; writing—review and editing: WW and KH. All authors contributed to the article and approved the submitted version.

Ethics approval and consent to participate

The study adhered to the Declaration of Helsinki or relevant guidelines and regulations, and was approved by the ethical review committee of the First Affiliated Hospital of Chongqing Medical University, Chongqing, China, with the ethics approval reference number 2021-648. Written informed consent to participate in this study was provided by the participants/next of kin.

For peer review only - http://bmjopen.bmj.com/site/about/guidelines.xhtml

Consent for publication

Not applicable.

Availability of data and materials

The datasets generated and/or analyzed during the current study are available from the corresponding author upon reasonable request.

Acknowledgments

We are indebted to KH and WJW from the First Affiliated Hospital of Chongqing Medical University, Chongqing, China for their help in supervising this manuscript.

For peer review only

For peer review only - http://bmjopen.bmj.com/site/about/guidelines.xhtml

References

[1] Cohen JE, Small C. Hypsographic demography: the distribution of human population by altitude. Proc Natl Acad Sci U S A. 1998;95(24):14009-14014.

[2] Wang GQ, Bai ZX, Shi J, Luo S, Chang HF, Sai XY. Prevalence and risk factors for eye diseases, blindness, and low vision in Lhasa, Tibet. Int J Ophthalmol. 2013;6(2):237-241. Published 2013 Apr 18.

[3] Narayanan DL, Saladi RN, Fox JL. Ultraviolet radiation and skin cancer. Int J Dermatol. 2010 Sep;49(9):978-86.

[4] Torii H, Kurihara T, Seko Y, Negishi K, Ohnuma K, Inaba T, Kawashima M, Jiang X, Kondo S, Miyauchi M, Miwa Y, Katada Y, Mori K, Kato K, Tsubota K, Goto H, Oda M, Hatori M, Tsubota K. Violet Light Exposure Can Be a Preventive Strategy Against Myopia Progression. EBioMedicine. 2017 Feb;15:210-219.

[5] Kobashi H, Yunoki S, Kato N, Shimazaki J, Ide T, Tsubota K. Evaluation of the Physiological Corneal Intrastromal Riboflavin Concentration and the Corneal Elastic Modulus After Violet Light Irradiation. Transl Vis Sci Technol. 2021;10(5):12.

[6] Althomali TA, Al-Qurashi IM, Al-Thagafi SM, Mohammed A, Almalki M. Prevalence of keratoconus among patients seeking laser vision correction in Taif area of Saudi Arabia. Saudi J Ophthalmol. 2018;32(2):114-118.

[7] Guo B, Lu P, Chen X, Zhang W, Chen R. Prevalence of dry eye disease in Mongolians at high altitude in China: the Henan eye study. Ophthalmic

For peer review only

Epidemiol. 2010;17(4):234-241.

[8] Jiachu D, Jin L, Jiang F, et al. Prevalence and service assessment of cataract in Tibetan areas of Sichuan Province, China: population-based study. BMJ Open. 2019 Nov 3;9(11):e031337.

[9] He H, Fu J, Meng Z, Chen W, Li L, Zhao X. Prevalence and associated risk factors for childhood strabismus in Lhasa, Tibet, China: a cross-sectional, school-based study. BMC Ophthalmol. 2020;20(1):463. Published 2020 Nov 25.

[10] Amit A, Gaurav K, Vikas A, Ashok K, Harpreet AS, Shivani A. Evaluation of intraocular pressure and corneal thickness in individuals at high altitude area (10000 ft above sea level). Rom J Ophthalmol. 2019;63(3):217-221

[11] Patyal S, Arora A, Yadav A, Sharma VK. Corneal Thickness in Highlanders. High Alt Med Biol. 2017;18(1):56-60.

[12] Wang W, Xiang Y, Zhu L, et al. Myopia progression and associated factors of refractive status in children and adolescents in Tibet and Chongqing during the COVID-19 pandemic. Front Public Health. 2022;10:993728. Published 2022 Oct 13.

[13] Mu J, Zeng D, Fan J, et al. The accuracy of the axial length and axial length/corneal radius ratio for myopia assessment among Chinese children. Front Pediatr. 2022;10:859944. Published 2022 Sep 6.

[14] Wilson S, Ctori I, Shah R, Suttle C, Conway ML. Systematic review and meta-analysis on the agreement of non-cycloplegic and cycloplegic refraction in children. Ophthalmic Physiol Opt. 2022 Nov;42(6):1276-1288.

For peer review only - http://bmjopen.bmj.com/site/about/guidelines.xhtml

[15] Baird PN, Saw SM, Lanca C, et al. Myopia. Nat Rev Dis Primers. 2020;6(1):99. Published 2020 Dec 17.

[16] Morgan IG, French AN, Ashby RS, et al. The epidemics of myopia: Aetiology and prevention. Prog Retin Eye Res. 2018;62:134-149.

[17] Qian X, Liu B, Wang J, et al. Prevalence of refractive errors in Tibetan adolescents. BMC Ophthalmol. 2018;18(1):118. Published 2018 May 11.

[18] Zhang J, Li Z, Ren J, et al. Prevalence of myopia: A large-scale population-based study among children and adolescents in weifang, china. Front Public Health. 2022;10:924566. Published 2022 Jul 25.

[19] Xu L, Zhuang Y, Zhang G, et al. Design, methodology, and baseline of whole city-million scale children and adolescents myopia survey (CAMS) in Wenzhou, China. Eye Vis (Lond). 2021;8(1):31. Published 2021 Aug 19.

[20] He X, Sankaridurg P, Naduvilath T, et al. Normative data and percentile curves for axial length and axial length/corneal curvature in Chinese children and adolescents aged 4-18 years. Br J Ophthalmol. 2023;107(2):167-175.

[21] Wu Q, Tian Q, Zhang X, Xu J, Tang G, Li R, Guo X, Xu Z, Feng J, Song J, Bi H. Prevalence of Refractive Error and Visual Acuity Among School Children in the Plateau Region of Qinghai, China. Int J Gen Med. 2021 Sep 16;14:5795-5805.

[22] Zhou X, Pardue MT, Iuvone PM, Qu J. Dopamine signaling and myopia development: What are the key challenges. Prog Retin Eye Res. 2017;61:60-71.

[23] Jiang, X., Pardue, M. T., Mori, K., Ikeda, S. I., Torii, H., D'Souza, S., Lang, R. A., Kurihara, T., & Tsubota, K. (2021). Violet light suppresses lens-induced

For peer review only - http://bmjopen.bmj.com/site/about/guidelines.xhtml

myopia via neuropsin (OPN5) in mice. Proceedings of the National Academy of Sciences of the United States of America, 118(22), e2018840118.

[24] Torii H, Ohnuma K, Kurihara T, Tsubota K, Negishi K. Violet Light Transmission is Related to Myopia Progression in Adult High Myopia. Sci Rep. 2017 Nov 6;7(1):14523.

[25] Zhang YY, Jiang WJ, Teng ZE, et al. Corneal curvature radius and associated factors in Chinese children: the Shandong Children Eye Study.PLoS One. 2015;10(2):e0117481. Published 2015 Feb 6.

[26] Xiong S, Zhang B, Hong Y, He X, Zhu J, Zou H, Xu X. The Associations of Lens Power With Age and Axial Length in Healthy Chinese Children and Adolescents Aged 6 to 18 Years. Invest Ophthalmol Vis Sci. 2017 Nov 1;58(13):5849-5855.

[27] Wang Y, Liu Y, Zhu X, Zhou X, He JC, Qu X. Corneal and lenticular biometry in Chinese children with myopia. Clin Exp Optom. 2022 Aug 31:1-9. .

[28] Metlapally R, Wildsoet CF. Scleral Mechanisms Underlying Ocular Growth and Myopia. Prog Mol Biol Transl Sci. 2015;134:241-8.

[29] Zhang D, Wang L, Jin L, Wen Y, Zhang X, Zhang L, Zhu H, Wang Z, Yu X, Xie C, Tong J, Shen Y. A Review of Intraocular Pressure (IOP) and Axial Myopia. J Ophthalmol. 2022 Jul 9;2022:5626479.

[30] Wang P, Chen S, Liu Y, Lin F, Song Y, Li T, Aung T, Zhang X; GSHM study group. Lowering Intraocular Pressure: A Potential Approach for Controlling High Myopia Progression. Invest Ophthalmol Vis Sci. 2021 Nov 1;62(14):17.

[31] Liu X, Pan X, Ma Y, Jin C, Wang B, Ning Y. Variation in intraocular pressure by

For peer review only - http://bmjopen.bmj.com/site/about/guidelines.xhtml

sex, age, and geographic location in China: A nationwide study of 284,937 adults. Front Endocrinol (Lausanne). 2022 Aug 25;13:949827.

[32] Hashemi H, Nabovati P, Khabazkhoob M, Emamian MH, Yekta A, Fotouhi A. Agreement of Central Corneal Thickness Measurements between Scheimpflug Photography and Optical Low-Coherence Reflectometry in Children. Semin Ophthalmol. 2020;35(4):252-256.

[33] Zheng Y, Huang G, Huang W, He M. Distribution of central and peripheral corneal thickness in Chinese children and adults: the Guangzhou twin eye study. Cornea. 2008;27(7):776-781.

[34] Zhou Q, Gao TY, Fan SJ, et al. Intraocular Pressure, Age, and Central Corneal Thickness in a Healthy Chinese Children Population: The Handan Offspring Myopia Study. Ophthalmic Epidemiol. 2022;29(5):499-506.

[35] Asharlous A, Hashemi H, Yekta A, et al. Topographic properties of the cornea in welders. Eur J Ophthalmol. 2022;32(1):165-169.

For peer review only

For peer review only - http://bmjopen.bmj.com/site/about/guidelines.xhtml

Table 1 Distribution of sample size and average altitude of each survey site

Groups		School	Sample Size (student)	Average altitude (meter)
Tibet	Karo District	Qamdo No. 1 Primary School	60	3250
		Qamdo Junior High School	30	
		Qamdo No. 1 Senior High School	120	
	Drayab County	Drayab County No. 1 Primary School	60	3170
		Drayab County Junior High School	30	
	Tengchen County	Xiexiong Township Central Primary School	60	3870
		Tengchen County Junior High School	30	
	Markam County	Qucainka Township Central Primary School	60	2300
		Mangkang County Junior High School	30	3865
	Total	/	480	/
Chongqing	Shapingba District	Fenghuang Experiental School	746	319
	Banan District	Banan Middle School	198	331
	Total	/	944	/

For peer review only

Table 2 Distribution of participants of different ages in Tibet and Chongqing groups

Age (year)	Tibet group			Chongqing group		
	Total	Males	Females	Total	Males	Females
**6**	22	10	12	29	15	14
**7**	38	17	21	59	24	35
**8**	36	20	16	69	39	30
**9**	32	20	12	55	22	33
**10**	39	19	20	53	34	19
**11**	42	19	23	78	39	39
**12**	53	34	19	86	40	46
**13**	49	18	31	79	35	44
**14**	44	25	19	74	49	25
**15**	38	20	18	66	35	31
**16**	28	11	17	61	13	48
**17**	19	10	9	37	9	28
**18**	8	3	5	2	2	0
**Total**	448	226	222	748	356	392

For peer review only

For peer review only - http://bmjopen.bmj.com/site/about/guidelines.xhtml

Table 3 Comparison of demographic data, myopia prevalence, and ocular biometric parameters in Tibetan and Chongqing children and adolescents

	Age	Tibet group	Chongqing group	*p* value	Adjusted *p*
**Sex Males(%)**	Total	226/448 (50.4%)	356/748 (47.6%)	0.339	
**Age (year)**	Total	12.09 ± 3.15	12.20 ± 3.10	0.549	
**Myopia prevalence (%)**	6-8	11/**96** (11.5%)	14/**157** (8.9%)	0.511	
	9-11	39/**113** (34.5%)	55/**186** (29.6%)	0.372	
	12-14	69/**146** (47.3%)	141/**239** (59%)	0.025	
	15-18	59/**93** (63.4%)	146/**166** (88%)	< 0.001	
	Total	178/**448** (39.7%)	356/**748** (47.6%)	0.008	
**SE (D)**	6-8	0 (-0.50, 0.38)	-0.13 (-0.50, 0.25)	0.191	
	9-11	-0.38 (-1.25, 0.13)	-0.44 (-1.13, 0)	0.829	
	12-14	-0.88 (-2.37, 0)	-1.5 (-3.13, -0.50)	0.001	
	15-18	-1.75 (-3.38, -0.88)	-3.63 (-4.88, -2.38)	< 0.001	
	Total	-0.63 (-2.00, 0.13)	-0.88 (-2.88, -0.13)	< 0.001	
**AL (mm)**	6-8	22.81 ± 0.76	22.94 ± 0.86	0.228	
	9-11	23.28 ± 0.85	23.57 ± 0.90	0.007	
	12-14	23.58 ± 1.01	24.17 ± 1.09	< 0.001	
	15-18	24.13 ± 1.03	24.86 ± 1.05	< 0.001	
	Total	23.45 ± 1.02	23.92 ± 1.19	< 0.001	
**Km (D)**	6-8	42.89 ± 1.49	43.24 ± 1.40	0.067	
	9-11	43.04 ± 1.34	43.17 ± 1.27	0.398	
	12-14	43.14 ± 1.34	43.30 ± 1.45	0.302	
	15-18	43.11 ± 1.49	43.32 ± 1.32	0.398	
	Total	43.06 ± 1.40	43.26 ± 1.36	0.014	
**AL/CR**	6-8	2.90 ± 0.08	2.94 ± 0.09	< 0.001	
	9-11	2.97 ± 0.10	3.01 ± 0.10	< 0.001	
	12-14	3.01 ± 0.12	3.10 ± 0.14	< 0.001	
	15-18	3.08 ± 0.13	3.19 ± 0.13	< 0.001	
	Total	2.99 ± 0.13	3.06 ± 0.15	< 0.001	
**ACD/AL**	6-8	0.132 ± 0.009	0.128 ± 0.010	0.003	
	9-11	0.136 ± 0.009	0.130 ± 0.009	< 0.001	
	12-14	0.135 ± 0.009	0.131 ± 0.010	< 0.001	
	15-18	0.134 ± 0.009	0.130 ± 0.009	< 0.001	
	Total	0.134 ± 0.009	0.130 ± 0.010	< 0.001	
**CCT (μm)**	6-8	541.35 ± 32.45	545.72 ± 30.08	0.278	
	9-11	540.26 ± 27.32	552.67 ± 32.32	0.001	
	12-14	530.19 ± 30.83	551.84 ± 29.48	< 0.001	
	15-18	533.37 ± 28.51	548.43 ± 31.35	< 0.001	
	Total	535.78 ± 30.16	550.01 ± 30.80	< 0.001	
**WTW (mm)**	6-8	11.68 ± 0.40	11.75 ± 0.36	0.156	0.328
	9-11	11.65 ± 0.35	11.73 ± 0.35	0.071	0.771
	12-14	11.57 ± 0.39	11.70 ± 0.38	0.002	0.079
	15-18	11.63 ± 0.36	11.69 ± 0.39	0.224	0.437
	Total	11.62 ± 0.38	11.71 ± 0.37	< 0.001	0.039
**ACD (mm)**	6-8	3.01 ± 0.22	2.94 ± 0.26	0.031	0.003
	9-11	3.16 ± 0.25	3.06 ± 0.27	0.003	< 0.001
	12-14	3.18 ± 0.26	3.18 ± 0.27	0.775	0.001
	15-18	3.25 ± 0.26	3.24 ± 0.27	0.867	< 0.001
	Total	3.15 ± 0.26	3.11 ± 0.29	0.015	< 0.001
**IOP (mmHg)**	6-8	15.30 ± 2.64	16.52 ± 3.28	0.001	0.004
	9-11	14.94 ± 3.05	16.52 ± 2.88	< 0.001	0.001
	12-14	14.23 ± 3.20	16.61 ± 2.83	< 0.001	< 0.001
	15-18	14.01 ± 2.59	16.01 ± 2.87	< 0.001	< 0.001
	Total	14.59 ± 2.96	16.43 ± 2.95	< 0.001	< 0.001
**Astig. F (D)**	Total	1.05 (0.70, 1.40)	1.10 (0.80, 1.60)	0.036	
**Astig. B (D)**	Total	0.30 (0.30, 0.40)	0.30 (0.30, 0.40)	0.326	

* Adjusted AL(WTW, ACD) or CCT (IOP)

For peer review only

For peer review only - http://bmjopen.bmj.com/site/about/guidelines.xhtml

SE, spherical equivalent; D, diopter; AL, axial length; AL/CR: AL to corneal radius of curvature ratio; IOP, intraoccular pressure, D, diopter; Km, mean curvature power of the cornea within the central 3-mm circle; Astig. F, astigmatism of the front surface; Astig. B, astigmatism of the back surface; CCT, corneal thickness at the apex; WTW, white to white distance; ACD, internal anterior chamber depth.

Table 4 The results of multiple linear regression analysis between SE and AL and Km

Group		AL			Km			Adjusted R^2
		B	Sta.β	*P*	B	Sta.β	*P*	
**Tibet group (n = 448)**	**SE**	-1.567	-0.880	< 0.001	-0.649	-0.500	< 0.001	0.651
**Chongqing Group (n = 748)**	**SE**	-1.616	-0.940	< 0.001	-0.768	-0.510	< 0.001	0.777
**Total (n = 1196)**	**SE**	-1.551	-0.905	< 0.001	-0.696	-0.486	< 0.001	0.728

B, Unstandardized Coefficient B, Sta.β, Standardized Coefficient Beta. AL, axial length; Km, mean curvature power of the cornea within the central 3-mm circle

Table 5 Comparison of ocular biometric parameters in altitude subgroups of primary school students

**Parameters**	**Group A n = 398**	**Group B n = 58**	**Group C n = 121**	**Group D n = 60**	*p*
**Sex Males(%)**	201 (50.5%)	31 (53.4%)	62 (51.2%)	31 (51.7%)	0.979
**Age (year)**	9.75 ± 1.85	9.87 ± 1.98	9.51 ± 2.03	9.83 ± 1.78	0.557
**Myopia prevalence**	99 (24.9%)	16 (27.6%)	39 (32.2%)	4 (6.7%)	0.002 (A,D: <0.05; B,D: <0.05; C,D: <0.05)
**SE (D)**	-0.38 (-0.88, 0.13)	-0.19 (-0.88, 0.25)	-0.25 (-1.25, 0.25)	-0.06 (-0.50, 0.25)	0.031 (A,D: 0.019)
**AL (mm)**	23.39 ± 0.98	23.19 ± 0.84	23.11 ± 0.90	22.97 ± 0.79	0.001 (A,C: 0.026; A,D: 0.007)
**IOP (mmHg)**	16.57 ± 3.05	16.12 ± 2.50	14.45 ± 3.05	15.17 ± 2.86	< 0.001 (A,C: <0.001; A,D: 0.004; B,C:0.004)
**AL/CR**	2.99 ± 0.11	2.94 ± 0.09	2.95 ± 0.11	2.92 ± 0.07	< 0.001 (A,B: 0.005; A,C: 0.001; A,D: <0.001)
**ACD/AL**	0.130 ± 0.01	0.136 ± 0.009	0.133 ± 0.009	0.134 ± 0.009	< 0.001 (A,B<0.001; A,C: 0.001; A,D: 0.015)
**Km (D)**	43.22 ± 1.34	42.87 ± 1.29	43.11 ± 1.46	42.93 ± 1.59	0.189
**Astig. F (D)**	1.10 (0.70, 1.50)	1.05 (0.70, 1.20)	1.20 (0.80, 1.60)	0.95 (0.80, 1.25)	0.041
**Astig. B (D)**	0.30 (0.20, 0.40)	0.30 (0.30, 0.40)	0.40 (0.30, 0.40)	0.30 (0.30, 0.40)	0.357
**CCT (μm)**	550.11 ± 31.2	533.17 ± 30.12	545.77 ± 28.13	532.4 ± 32.50	< 0.001 (A,B: 0.001; A,D: <0.001; C,D: 0.036)
**WTW (mm)**	11.73 ± 0.36	11.63 ± 0.36	11.63 ± 0.38	11.71 ± 0.42	0.024 (A,C: 0.048)
**ACD (mm)**	3.03 ± 0.27	3.16 ± 0.27	3.08 ± 0.24	3.07 ± 0.25	0.004 (A,B: 0.004)

SE, spherical equivalent; D, diopter; AL, axial length; AL/CR: AL to corneal radius of curvature ratio; IOP, intraoccular pressure, D, diopter; Km, mean curvature power of the cornea; Astig. F, astigmatism of the front surface; Astig. B, astigmatism of the

For peer review only - http://bmjopen.bmj.com/site/about/guidelines.xhtml

back surface; CCT, corneal thickness at the apex; WTW, white to white distance; ACD, internal anterior chamber depth.

For peer review only

For peer review only - http://bmjopen.bmj.com/site/about/guidelines.xhtml

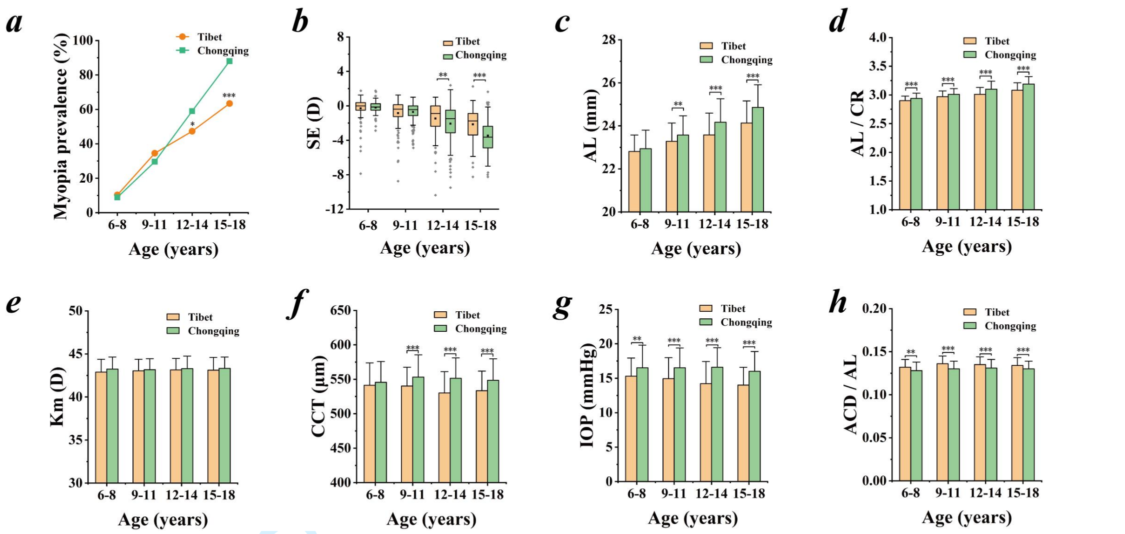

Figure S1 Comparison of myopia prevalence (a), SE (b), AL (c), AL/CR (d), Km (e), CCT (f), IOP (g), and ACD/AL (h) in Tibetan and Chongqing children and adolescents by age groups. SE, spherical equivalent; D, diopter; AL, axial length; AL/CR, AL to corneal radius of curvature ratio; Km, mean curvature power of the cornea; CCT, corneal thickness at the apex;IOP, intraoccular pressure; ACD, internal anterior chamber depth.

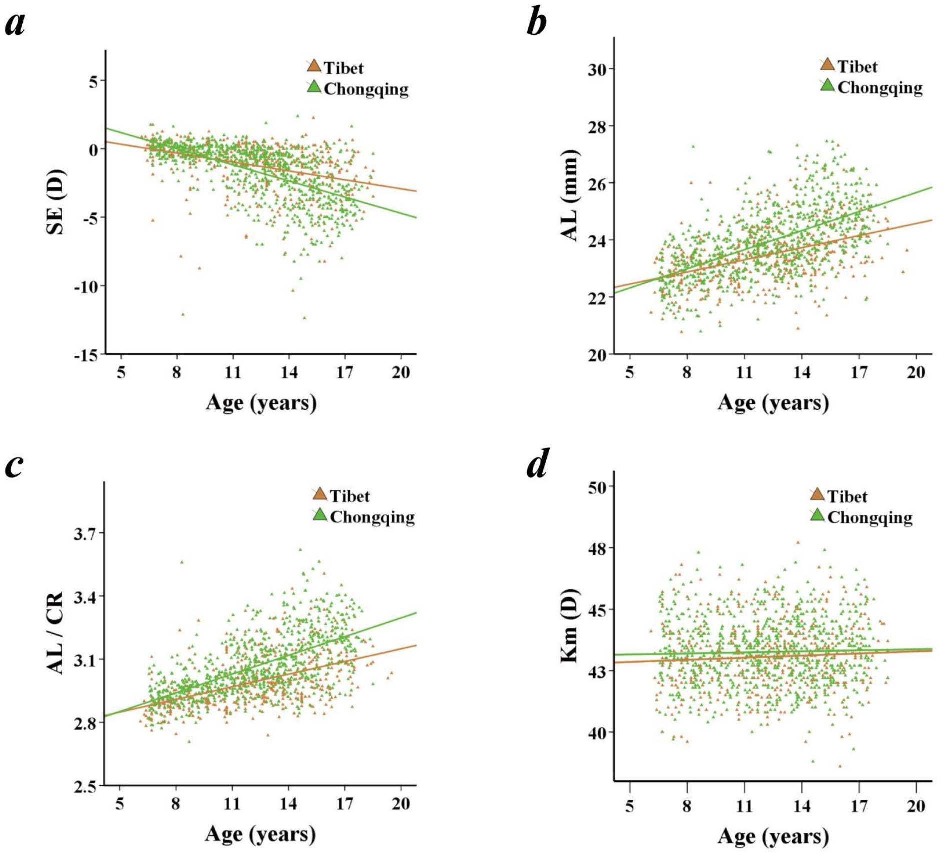

Figure S2 Linear regression model of SE (a), AL (b), AL/CR (c) and Km (d) with age. SE, spherical equivalent; D, diopter; AL, axial length; AL/CR, AL to corneal radius of curvature ratio; Km, mean curvature power of the cornea.

For peer review only - http://bmjopen.bmj.com/site/about/guidelines.xhtml

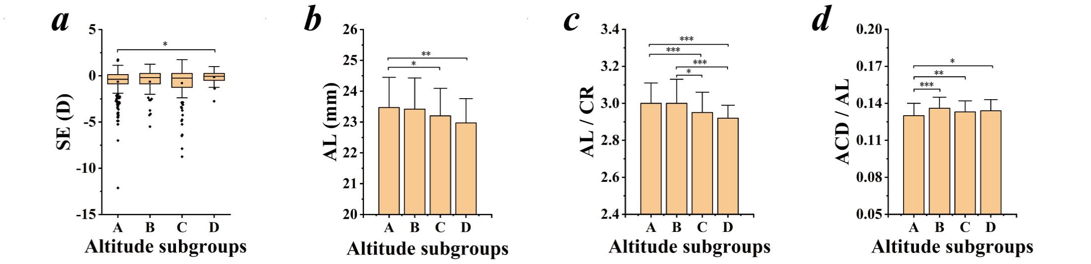

Figure S3 Comparison of SE (a), AL (b), AL/CR (c) and ACD/AL (d) in altitude subgroups of primary school students. SE, spherical equivalent; D, diopter; AL, axial length; AL/CR, AL to corneal radius of curvature ratio; ACD, internal anterior chamber depth. Group A, with an average altitude of 325 meters; Group B, with an average altitude of 2,300 meters; Group C, with an average altitude of 3,250 and 3,170 meters, Group D, with an average altitude of 3,870 meters.

For peer review only

STROBE Statement—Checklist of items that should be included in reports of ***cross-sectional studies***

	Item No	Recommendation	Page No
**Title and abstract**	1	(*a*) Indicate the study's design with a commonly used term in the title or the abstract	1
		(*b*) Provide in the abstract an informative and balanced summary of what was done and what was found	1
**Introduction**			
Background/rationale	2	Explain the scientific background and rationale for the investigation being reported	3
Objectives	3	State specific objectives, including any prespecified hypotheses	5
**Methods**			
Study design	4	Present key elements of study design early in the paper	5
Setting	5	Describe the setting, locations, and relevant dates, including periods of recruitment, exposure, follow-up, and data collection	5
Participants	6	(*a*) Give the eligibility criteria, and the sources and methods of selection of participants	5
Variables	7	Clearly define all outcomes, exposures, predictors, potential confounders, and effect modifiers. Give diagnostic criteria, if applicable	6
Data sources/ measurement	8*	For each variable of interest, give sources of data and details of methods of assessment (measurement). Describe comparability of assessment methods if there is more than one group	
Bias	9	Describe any efforts to address potential sources of bias	6
Study size	10	Explain how the study size was arrived at	6
Quantitative variables	11	Explain how quantitative variables were handled in the analyses. If applicable, describe which groupings were chosen and why	6
Statistical methods	12	(*a*) Describe all statistical methods, including those used to control for confounding	7
		(*b*) Describe any methods used to examine subgroups and interactions	7
		(*c*) Explain how missing data were addressed	7
		(*d*) If applicable, describe analytical methods taking account of sampling strategy	7
		(*e*) Describe any sensitivity analyses	
**Results**			
Participants	13*	(a) Report numbers of individuals at each stage of study—eg numbers potentially eligible, examined for eligibility, confirmed eligible, included in the study, completing follow-up, and analysed	7
		(b) Give reasons for non-participation at each stage	
		(c) Consider use of a flow diagram	
7Descriptive data	14*	(a) Give characteristics of study participants (eg demographic, clinical, social) and information on exposures and potential confounders	
		(b) Indicate number of participants with missing data for each variable of interest	
Outcome data	15*	Report numbers of outcome events or summary measures	
Main results	16	(*a*) Give unadjusted estimates and, if applicable, confounder-adjusted estimates and their precision (eg, 95% confidence interval). Make clear which confounders were adjusted for and why they were included	8

For peer review only

		(*b*) Report category boundaries when continuous variables were categorized	
		(*c*) If relevant, consider translating estimates of relative risk into absolute risk for a meaningful time period	
Other analyses	17	Report other analyses done—eg analyses of subgroups and interactions, and sensitivity analyses	9
**Discussion**			
Key results	18	Summarise key results with reference to study objectives	10
Limitations	19	Discuss limitations of the study, taking into account sources of potential bias or imprecision. Discuss both direction and magnitude of any potential bias	14
Interpretation	20	Give a cautious overall interpretation of results considering objectives, limitations, multiplicity of analyses, results from similar studies, and other relevant evidence	14
Generalisability	21	Discuss the generalisability (external validity) of the study results	14
**Other information**			
Funding	22	Give the source of funding and the role of the funders for the present study and, if applicable, for the original study on which the present article is based	15

*Give information separately for exposed and unexposed groups.

Note: An Explanation and Elaboration article discusses each checklist item and gives methodological background and published examples of transparent reporting. The STROBE checklist is best used in conjunction with this article (freely available on the Web sites of PLoS Medicine at http://www.plosmedicine.org/, Annals of Internal Medicine at http://www.annals.org/, and Epidemiology at http://www.epidem.com/). Information on the STROBE Initiative is available at www.strobe-statement.org.

For peer review only
